# Cysteine Peptidase Cathepsin X as a Therapeutic Target for Simultaneous TLR3/4-mediated Microglia Activation

**DOI:** 10.1007/s12035-021-02694-2

**Published:** 2022-01-23

**Authors:** Anja Pišlar, Biljana Božić Nedeljković, Mina Perić, Tanja Jakoš, Nace Zidar, Janko Kos

**Affiliations:** 1 Department of Pharmaceutical Biology, Faculty of Pharmacy, University of Ljubljana, Aškerčeva 7, 1000 Ljubljana, Slovenia; 2 Institute for Physiology and Biochemistry, Faculty of Biology, University of Belgrade, Studentski trg 16, 11000 Belgrade, Serbia; 3 Department of Pharmaceutical Chemistry, Faculty of Pharmacy, University of Ljubljana, Aškerčeva 7, 1000 Ljubljana, Slovenia; 4 Department of Biotechnology, Jožef Stefan Institute, Jamova cesta 39, 1000 Ljubljana, Slovenia

**Keywords:** Microglia, Toll-like receptors, Cathepsin X, Proinflammatory mediators, Neuroinflammation, Neuroprotection

## Abstract

Microglia are resident macrophages in the central nervous system that are involved in immune responses driven by Toll-like receptors (TLRs). Microglia-mediated inflammation can lead to central nervous system disorders, and more than one TLR might be involved in these pathological processes. The cysteine peptidase cathepsin X has been recognized as a pathogenic factor for inflammation-induced neurodegeneration. Here, we hypothesized that simultaneous TLR3 and TLR4 activation induces synergized microglia responses and that these phenotype changes affect cathepsin X expression and activity. Murine microglia BV2 cells and primary murine microglia were exposed to the TLR3 ligand polyinosinic-polycytidylic acid (poly(I:C)) and the TLR4 ligand lipopolysaccharide (LPS), individually and simultaneously. TLR3 and TLR4 co-activation resulted in increased inflammatory responses compared to individual TLR activation, where poly(I:C) and LPS induced distinct patterns of proinflammatory factors together with different patterns of cathepsin X expression and activity. TLR co-activation decreased intracellular cathepsin X activity and increased cathepsin X localization at the plasma membrane with concomitant increased extracellular cathepsin X protein levels and activity. Inhibition of cathepsin X in BV2 cells by AMS36, cathepsin X inhibitor, significantly reduced the poly(I:C)- and LPS-induced production of proinflammatory cytokines as well as apoptosis. Additionally, inhibiting the TLR3 and TLR4 common signaling pathway, PI3K, with LY294002 reduced the inflammatory responses of the poly(I:C)- and LPS-activated microglia and recovered cathepsin X activity. We here provide evidence that microglial cathepsin X strengthens microglia activation and leads to subsequent inflammation-induced neurodegeneration. As such, cathepsin X represents a therapeutic target for treating neurodegenerative diseases related to excess inflammation.

**Supplementary Information:**

The online version contains supplementary material available at 10.1007/s12035-021-02694-2.

## Introduction

Innate immune pathways are early responses that are important for pathogen control. They can be activated by specific pattern recognition receptors that recognize pathogen-associated molecule patterns (PAMPs) or danger-associated molecule patterns [1–3]. In the central nervous system (CNS), microglia are resident innate immune cells that provide the first line of defense against pathogens [4]. In the steady-state, microglia secrete neurotrophic factors that regulate normal brain functions. Upon pathogen recognition, homeostatic microglia are transformed into their activated phenotype, which can lead to neuronal tissue damage and the expression of genes related to neuroinflammation [5–7]. This phenotype includes the expression of the cell surface receptors, which make these cells highly reactive to a variety of stimuli [2, 8]. Several pattern recognition receptors have been described, including Toll-like receptors (TLRs), C-type lectins, cytoplasmic retinoic acid-inducible gene-I-like receptors, and nucleotide oligomerization domain-like receptors [9–11]. The evolutionarily conserved TLRs are the best characterized pattern recognition receptors, and they can initiate long-standing and protective immunity through presenting antigen motifs to the cells of the adaptive immune system. To date, 10 functionally active TLRs have been identified in humans, and 12 in mice. These are most commonly divided into two groups according to their cellular localization and PAMP ligands [12]. The PAMPs that are frequently used to study various activation pathways of antigen-presenting cells, including microglia, are lipopolysaccharide (LPS) and polyinosinic-polycytidylic acid (poly(I:C)) [13, 14]. LPS is a major component of the cell wall of Gram-negative bacteria and is recognized by a receptor complex that consists of TLR4/myeloid differentiation protein 2 and CD14 [15–17]. Conversely, poly(I:C) is a synthetic analog of double-stranded RNA and can be generated during the replication of RNA and DNA viruses [18]. This PAMP is mainly recognized by the TLR3 receptor [19, 20]. Stimulating the TLRs on murine microglia by these PAMPs initiates signaling cascades that ultimately lead to the activation of nuclear factor kappa B (NF-κB) [19, 21]. This, in turn, induces the production of proinflammatory cytokines, such as tumor necrosis factor (TNF)-α and interleukin (IL)-6 [3, 22].

In addition to proinflammatory cytokines, activated microglia also synthesize and secrete lysosomal peptidases [23–31]. This especially occurs at the early stages of inflammation and triggers signaling pathways in a pathological cascade that aggravates neuroinflammation. Exposure to TLR4 agonist LPS leads to an increase in cathepsins B, L, K, S, H, and X [26, 28, 31]. Additionally, cathepsins have been linked with regulating TLR3, which is processed by cathepsins within Loop1 of leucine-rich repeat 12. When proteolytic cleavage is inhibited by either a cathepsin inhibitor or Loop1 deletion, TLR3 can still be activated by poly(I:C) in many types of cell lines that express transiently transfected TLR3 [32]. Among cathepsins, cysteine cathepsin X has been intensively studied in association with TLR4 agonist-induced neuroinflammation [26, 31, 33, 34], whereas the association with TLR3 is unknown.

Cathepsin X is a carboxypeptidase expressed predominantly in immune cells [35] but also in brain cells [30, 36]. In recent years, an increasing number of studies have linked increased cathepsin X expression and activity in brain cells with neurodegenerative processes in the CNS. A comprehensive comparative gene expression analysis of murine models of CNS disorders, i.e., Alzheimer’s disease, multiple sclerosis, and stroke, revealed that the cathepsin X gene *Ctsz* is one of the 18 genes that show increased expression [37]. High levels of cathepsin X mRNA and protein have been reported for glial cells of degenerating brain regions in the transgenic murine models of amyotrophic lateral sclerosis and Alzheimer’s disease as well as around senile plaques in the brains of Alzheimer’s disease patients [30, 36]. In a rat brain with Alzheimer’s disease pathology, cathepsin X upregulation was restricted to the microglia surrounding amyloid plaques in which it co-localized with its target, γ-enolase [30]. Additionally, cathepsin X is disproportionately expressed and secreted by microglia and astrocytes in response to neuronal damage and inflammatory stimulus, both in vitro and in vivo [26, 31, 38, 39]. Furthermore, dendritic cells in the brain of older mice have increased cathepsin X protein levels that correlate with markers of neuroinflammation [33]. Allan et al. showed that cathepsin X-deficient mice have reduced neuroinflammation-related markers and particularly low circulating levels of IL-1β during experimental autoimmune encephalomyelitis [40].

The significant role of cathepsin X in neuroinflammation has been further demonstrated with the irreversible cathepsin X inhibitor AMS36, which suppressed proinflammatory molecule production and attenuated cytokine release from activated microglia, thus also reducing microglia-mediated neurotoxicity [31]. In vivo studies have shown that unilateral intrastriatal injection of LPS induces strong upregulation of cathepsin X expression and activity in the ipsilateral striatum, which is predominately restricted to activated microglia and reactive astrocytes. Additionally, continuous administration of AMS36 along with LPS injection indicated moderate protective effects against LPS-induced striatal degeneration [34]. These data indicate that cathepsin X is a key player in microglia-mediated neuroinflammation; however, its mode of action in microglia remains unclear.

Although both LPS and poly(I:C) trigger inflammatory responses, the intracellular signaling pathways and patterns of proinflammatory cytokines triggered by their corresponding TLRs may differ [13] and has not been yet studied as other pairwise stimulation of TLRs [41]. Furthermore, to the best of our knowledge, no reports have investigated cathepsin X activity during the proinflammatory responses in microglia induced by simultaneous stimulation with LPS and poly(I:C). Therefore, in the present study, we compared first the impact of sole and pairwise TLR3 and TLR4 activation by their respective ligands, poly(I:C) and LPS on microglia activation, and further evaluated cathepsin X expression patterns and cellular localization, in response to TLR3/TLR4 co-activation. As such, we have addressed the involvement of cathepsin X in the underlying mechanisms of LPS/poly(I:C)-induced microglia inflammation.

## Materials and Methods

### Ethical approval

The experimental procedures performed on the animals for the establishment of primary murine microglia were approved by the Ethics Review Committee for Animal Experimentation of the Faculty of Biology, University of Belgrade, and Ministry of Agriculture and Environmental Protection, Republic of Serbia, Veterinary Directorate No. 323–07-618,012,019–05.

### Cell Cultures

The murine microglia BV2 cells were a generous gift from Dr. Alba Minelli (University of Perugia, Perugia, Italy). The SH-SY5Y human neuroblastoma cells were purchased from American Type Culture Collection (ATCC CRL-2266; VA, USA). The BV2 and SH-SY5Y cells were cultured in Dulbecco’s modified Eagle’s medium (Sigma-Aldrich, St. Louis, MO, USA) supplemented with 10% fetal bovine serum (HyClone, UT, USA), 2 mM l-glutamine, 50 U/mL penicillin, and 50 μg/mL streptomycin (Sigma-Aldrich). The cells were maintained at 37 °C in a humidified atmosphere of 95% air and 5% CO_2_ and grown to 80% confluence. These cells were subcultured two or three times per week using 0.25% trypsin (Sigma-Aldrich).

### Primary Murine Microglia Isolation

Primary mixed glial cultures were prepared from postnatal day 2 C57BL/6 mice pups, as previously described [42] with some adjustment. In brief, brain cortices from 2 to 3 pups were isolated in cold phosphate-buffered saline (PBS), and then the tissue was minced and digested in 1 mg/mL trypsin (Sigma-Aldrich, USA) at 37 °C for 10 min. After digestion, complete culture media was added, consisting of Dulbecco’s modified Eagle’s medium (Thermo Fischer Scientific, USA), supplemented with 10% fetal bovine serum (Thermo Fischer Scientific, USA), D-glucose up to a final concentration of 25 mM (Sigma-Aldrich, USA), 100 U/mL penicillin, and 100 µg/mL streptomycin (Thermo Fischer Scientific, USA); the tissue was centrifuged at 500 × *g* for 8 min. The pellet was resuspended in complete culture medium, passed through needles of decreasing diameter, and centrifuged at 500 × *g* for 8 min. The pellet was resuspended in complete culture medium, and the cells were cultured in flasks and maintained at 37 °C in a humidified 5% CO_2_ atmosphere. Every third day, 50% of the existing medium was replaced with fresh medium. Within 10–14 days after plating, mixed glial cultures formed confluent monolayers with microglia cells growing on top. To obtain pure cultures of microglia from these initial co-cultures, flasks with cultivated cells were shaken for 2 h at 250 rpm on an orbital shaker at 37 °C. The medium containing the detached microglia was collected and centrifuged at 500 × *g* for 5 min. The supernatants were replaced with fresh culture medium, and the resuspended microglia were seeded into subcultures. The flasks were refilled with culture medium, and microglia harvesting was repeated after 5 days. For the experiments with these purified microglia, the cells were seeded as subcultures in 24-well culture plates (2 × 10^4^ cells/mL).

### Cell Treatments

BV2 microglia and primary murine microglia were stimulated with 1 µg/mL LPS (L6529; *Escherichia coli* 055:B5; Sigma-Aldrich) and/or 10 µg/mL poly(I:C) (Sigma-Aldrich) in serum-free medium for 24 h, unless otherwise indicated. Successful activation was checked routinely by measuring nitrite in the culture supernatants with Griess reagent kits, as described below.

Additionally, microglia were pre-treated 1 h prior to LPS and/or poly(I:C) stimulation with the following experimental agents: the specific, irreversible inhibitor of cathepsin X AMS36 (10 µM; synthesized as reported [43, 44]); the phosphoinositide 3 kinase (PI3K) inhibitors LY294002 (10 µM; Sigma-Aldrich) or wortmannin (1 µM; Sigma-Aldrich); the pan-caspase inhibitor carbobenzoxy-valyl-alanyl-aspartyl-[O-methyl]-fluoromethylketone (Z-VAD-FMK; 10 µM; Bachem, Switzerland); the cathepsin B inhibitor *N*-[[(2*S*,3*S*)-3-[(propylamino)carbonyl]-2-oxiranyl]carbonyl]-L-isoleucyl-L-proline (CA-074; 10 µM; Bachem); the cathepsin L inhibitor (from the Katunuma (CLIK) series) CLIK-148 (1 µM; a generous gift from Prof. Nobuhiko Katunuma, University of Tokushima, Tokushima, Japan); or the cathepsin S inhibitor leucine-homophenylalanine-vinyl phenyl sulfone (LHVS; 10 µM; a generous gift from Dr. James McKerrow, University of California, San Francisco, CA, USA). The experiments were generally terminated within 24 h of these treatments.

After stimulation, the microglia were harvested and further processed for ELISA, enzyme activity assays, immunofluorescence staining, flow cytometry, and western blotting. For co-cultures, the supernatants from the microglia were collected, centrifuged at 1200 rpm for 5 min to remove any cellular material, and transferred to cultured SH-SY5Y neuroblastoma cells.

### Microglia Culture Supernatant Transfer Model

To investigate the effects of potential soluble factors secreted by activated microglia, SH-SY5Y cells were treated with the supernatants from microglia BV2 cells stimulated for 24 h with 1 µg/mL LPS and/or 10 µg/mL poly(I:C) in the absence or presence of 10 µM AMS36 (pre-treatment). The SH-SY5Y cells were examined for neuronal viability 24 h and 48 h after transferring the microglia culture supernatant.

### Cell Viability

Cell viability was evaluated using the [3-(4,5-dimethylthiazol-2-yl)-5-(3-carboxymethoxyphenyl)-2-(4-sulfophenyl)-2H-tetrazolium (MTS) assay. The SH-SY5Y cells were seeded into 96-well culture plates with complete medium in quadruplicate (2 × 10^4^ cells/mL). The next day, the cells were treated as described above, and after the times indicated, the cell viability was determined using the CellTiter 96 Aqueous One Solution Cell Proliferation Assay (Promega, WI, USA) in accordance with the manufacturer’s instructions. Absorbance was measured at 490 nm with an automatic microplate reader (Tecan Safire^2^, Switzerland). Data are expressed as proportions (%) relative to the untreated control cells.

### Transfection

Cathepsin X was silenced using small interfering RNA (siRNA) oligonucleotides that target cathepsin X mRNA (siRNA-CatX; sc-44661; Santa Cruz Biotechnology, TX, USA). The BV2 cells were transiently transfected with siRNA-CatX using Lipofectamine 2000 (Invitrogen, CA, USA), according to the manufacturer’s protocol. Briefly, the BV2 cells were seeded into 12-well culture plates (5 × 10^4^ cells/well) in 1 mL growth medium without antibiotics. For transfection, Lipofectamine 2000 was gently mixed before use, diluted in Opti-MEM I medium without serum (Invitrogen), and left for 5 min at room temperature (RT). The diluted Lipofectamine 2000 was then combined with the diluted siRNA oligomer (to 40 nM RNA) in Opti-MEM I medium, gently mixed, and left for a further 20 min at RT. Next, 200 µL of transfection complex was added to each of the wells, containing BV2 cells and medium, and incubated at 37 °C in a humidified atmosphere of 95% air and 5% CO_2_ for 6 h. Afterwards, the medium with the transfection complex was replaced with complete growth medium for 24 h. Control siRNA (siRNA-Ctrl; sc-37007; Santa Cruz Biotechnology) was used as the negative transfection control. The effects of siRNA silencing after 48 h were analyzed by flow cytometry, as described previously [45], using a specific cathepsin X antibody (AF934; R&D Systems, MN, USA).

### Quantification of Nitrite

After the LPS and/or poly(I:C) stimulation described above, aliquots of the cell supernatants were obtained by centrifugation at 1200 rpm for 5 min and were then either stored at – 80 °C or used immediately to determine the nitrite content, an indicator of nitric oxide (NO) production [46]. The accumulation of NO in culture supernatants was determined using Griess reagent kits (Promega), according to the manufacturer’s instructions. Absorbance was measured at 550 nm using an automatic microplate reader (Tecan Safire^2^). NaNO_2_ was used as the standard to calculate nitrite concentrations (in µM).

### Cytokine Assay

Cells were centrifuged at 1200 rpm for 5 min, and the culture supernatants were either stored at − 80 °C or used immediately to measure the released cytokines. Cytokine production was determined by flow cytometry using cytometric bead arrays: mouse inflammatory cytokine kits containing beads for IL-2, IL-4, IL-6, IL-10, IL-17A, interferon-γ (IFN-γ), and TNF-α (BD Biosciences, CA, USA). A flow cytometer (FACS Calibur, BD Biosciences) with a four-color sorting option and the CELLQuest software (BD Biosciences) was used. Standard curves generated using the recombinant cytokines provided in the kits (0–5000 pg/mL) were used to define the cytokine concentrations (in pg/mL). Data were analyzed using the FlowJo software (Tree Star, Inc., OR, USA).

### ELISA

Culture supernatants and cell lysates were prepared for the analysis of cathepsin X protein levels. BV2 cells were seeded into 6-well culture plates (5 × 10^5^ cells/mL) and stimulated as described above. Primary murine microglia isolation and stimulation were performed as described above. The microglia culture supernatants were then collected, centrifuged, and stored at – 80 °C. The cells were washed with PBS (pH 7.4), harvested in cell lysis buffer (0.05 M sodium acetate buffer, pH 5.5, 1 mM EDTA, 0.1 M NaCl, 0.25% Triton X-100), and stored at – 80 °C. Total protein concentrations were determined using assay kits (DC Protein Assay; Bio-Rad, CA, USA). Cathepsin X protein levels were determined by ELISA, as reported previously [35], using a goat anti-cathepsin X antibody (AF934; R&D Systems) as the capture antibody and a mouse anti-cathepsin X monoclonal antibody (3B10; produced in-house) conjugated with horseradish peroxidase as the detection antibody. The amount of cleaved 3,3’,5,5’-tetramethylbenzidine substrate (Bio-Rad) was determined by measuring the absorbance at 450 nm, and the cathepsin X protein levels are expressed relative to those in the untreated control cells.

#### Cathepsin X Activity

Cathepsin X activity in cell lysates and culture supernatants of activated microglia were measured using the cathepsin-X-specific, intramolecularly quenched fluorogenic substrate Abz-Phe-Glu-Lys(Dnp)-OH (Jiangsu Vcare Pharmatech Co., China), as described previously [44]. Briefly, the BV2 cells were seeded into 6-well culture plates (5 × 10^5^ cells/mL), and primary murine microglia were isolated and stimulated as described above. After stimulation, the culture supernatants were collected, centrifuged, and stored at – 80 °C until use. Cell lysates were prepared using cell lysis buffer (0.05 M sodium acetate, pH 5.5, 1 mM EDTA, 0.1 M NaCl, 0.25% Triton X-100), and the protein concentrations were determined as indicated above. The following was mixed and incubated at 37 °C for 10 min: microglia culture supernatant (100 µL), activation buffer (150 µL; 100 mM sodium acetate, pH 5.5, 0.1% polyethylene glycol 8000, 5 mM dithiothreitol, 1.5 mM EDTA), and lysate protein (50 µg in 200 µL activation buffer). Next, cathepsin X activity was measured using 10 µM Abz-Phe-Glu-Lys(Dnp)-OH. The fluorometric reaction was quantified at 37 °C at an excitation wavelength of 320 nm and emission wavelength of 420 nm with a microplate reader (Tecan Safire^2^). Data are presented as changes in fluorescence as a function of time (ΔF/Δt), and cathepsin X activity is expressed relative to that of the untreated control cells.

#### RNA Isolation and Quantitative Real-Time PCR Analysis

Total RNA was extracted from BV2 cells using the RiboZol reagent (VWR Life Science) and purified using 5Prime Phase Lock Gel tubes (Qunatabio) before ethanol precipitation. Total RNA (1 µg) was reverse transcribed using oligo(dT)18 primers with the RevertAid First Strand cDNA Synthesis Kit (Thermo Scientific) according to manufacturer’s instructions. qPCR was performed using 0.6 ng of cDNA per 10 µL of reaction with the Maxima SYBR Green/ROX qPCR Master Mix 2X (Thermo Scientific) on LightCycler 480 (Roche) under the following conditions: 10 min at 95 °C; 40 cycles of denaturation at 95 °C, 15 s, and annealing at 60 °C, 1 min. Primers (Table 1) were validated by melting curve analysis, and reaction efficiencies were confirmed to be within 90–110%. Among the three reference genes tested (ACTB, RPLP0, and ATP5B), RPLP0 was selected for normalization by NormFinder [47]. Relative gene expression was calculated according to the 2^–∆∆Ct^ method.

**Table 1:** List of primers used for quantitative real-time PCR analysis

Gene	Primer pair	Sequence (5′ → 3′)
*CTSZ*	F R	ACCAGGCCGTTATCAACCACA CATCCAGCCTTTCTCACCCCAG
*ACTB*	F R	GGCTGTATTCCCCTCCATCG CCAGTTGGTAACAATGCCATGT
*RPLP0*	F R	AGATTCGGGATATGCTGTTGGC TCGGGTCCTAGACCAGTGTTC
*ATP5B*	F R	ACCACCACCAAGAAGGGATCG GGGTCAGTCAGGTCATCAGCA

#### Immunofluorescence Staining

Next, CD11b expression and co-localization of cathepsin X with the lysosomal marker LAMP1 and the plasma membrane marker cadherin were investigated. BV2 cells were cultured on glass coverslips (2 × 10^4^ cells/mL) in 24-well culture plates; the next day, they were stimulated as described above. The cells were then fixed with 5% formalin in PBS at RT for 30 min and permeabilized with 0.05% Tween 20 in PBS for 10 min. Nonspecific staining was blocked with 3% bovine serum albumin in PBS (pH 7.4) for 30 min. The cells were then incubated with mouse anti-CD11b (1:100; Abcam, UK), goat anti-cathepsin X (1:500; R&D System), rabbit anti-LAMP1 (1:100; Sigma-Aldrich), or rabbit anti-cadherin (1:1000; Abcam) antibodies in blocking buffer for 2 h at RT. The cells were washed with PBS and further incubated for an additional 1.5 h with Alexa 488- and Alexa 555-labeled secondary antibodies (Molecular Probes, OR, USA). After washing with PBS, the ProLong Gold Antifade Mountant with DAPI (Invitrogen) was used to mount the coverslips onto glass slides. Fluorescence microscopy was performed using a confocal microscope (LSM 710; Carl Zeiss, Germany) with the ZEN 2011 image software. For each treatment, more than 10 images were taken, with cells selected at random and the relative co-localization areas were analyzed. Quantification of the co-localization areas is presented as the average pixels in the third quadrant in the scatter plot of the two fluorescence intensities (*n* ≥ 10). All images were captured under the same exposure time settings. To improve the signal/noise ratio, 4 frames/image were averaged. Secondary antibody controls were also performed in parallel with each experiment (data not shown).

#### Western Blotting

The protein levels of inducible nitric oxide synthase (iNOS) were determined by Western blotting, as described previously [48]. Briefly, the BV2 cells were seeded into 6-well culture plates (5 × 10^5^ cells/mL). After stimulation, cell lysates were prepared using cell lysis buffer (50 mM HEPES (pH 6.5) 150 mM NaCl, 1 mM EDTA, 1% Triton X-100) supplemented with a cocktail of protease and phosphatase inhibitors. The protein concentrations were determined as indicated above. The separated proteins were transferred to nitrocellulose membranes and immunoblotted with rabbit anti-iNOS (1:500; Abcam) and mouse anti-β-actin (1:500; Sigma-Aldrich) antibodies. Signals from the horseradish-peroxidase-conjugated anti-rabbit (1:3000; Cell Signaling Technology, MA, USA) and anti-mouse (1:5000, Merck Millipore, MA, USA) secondary antibodies were visualized using enhanced chemiluminescence detection kits (Thermo Fisher Scientific, IL, USA). The band intensities were quantified using the Gene Tools software (Sygene, UK), and the data are expressed relative to the controls.

#### Staining with Propidium Iodide

Cytotoxicity was determined using the propidium iodide fluorescence method. The BV2 cells were seeded into 24-well culture plates (5 × 10^4^ cells/well); the next day, they were treated as described above. After the 24 h treatments, the cells were labeled with 30 µM propidium iodide (Invitrogen) for an additional 30 min at 37 °C. The cells were then analyzed for cytotoxicity by flow cytometry (Attune NxT; Thermo Fisher Scientific). The proportions (%) of propidium-iodide-positive cells were evaluated using FlowJo software, and data are presented relative to the controls.

#### Detection of Apoptosis

Apoptosis was detected and quantified using the Annexin-FITC Apoptosis Detection Kit (Sigma-Aldrich), according to the manufacturer’s instructions. Briefly, the BV2 cells were seeded into 24-well culture plates (5 × 10^4^ cells/well); the next day, they were treated as described above. Following stimulation, the cells were washed with cold PBS and resuspended in 500 µL of binding buffer. Then, 5 µL of FITC-labeled annexin V and 10 µL of propidium iodide were added to the cells, which were incubated for 15 min in the dark. Cell apoptosis was analyzed using a flow cytometer (FACS Calibur; BD Bioscience). The proportions (%) of apoptotic (annexin-V-positive) cells were determined using FlowJo software.

#### Caspase 3/7 Activity Assay

The activities of caspases 3 and 7 were determined in total cell lysates of the BV2 cells stimulated as described above. These cells were seeded into 6-well culture plates (5 × 10^5^ cells/mL). After stimulation, the cell lysates were prepared, and the caspase 3/7 activities were measured using a fluorescent peptide substrate (N-acetyl-L-α-aspartyl-L-α-glutamyl-L-valyl-N-[2-oxo-4-(trifluoromethyl)-2H-1-benzopyran-7-yl]-L-α-asparagine [Ac-DEVD-AFC]; Bachem), as described [49]. Fluorescence was monitored continuously for 30 min at an excitation wavelength of 405 nm and an emission wavelength of 535 nm using a fluorescence microplate reader (Tecan Safire^2^). Data are changes in fluorescence as a function of time (ΔF/Δt); caspase 3/7 activities are expressed as DEVDase activities relative to the untreated control cells.

#### Flow Cytometry Analysis of Phosphorylated Proteins

To determine the active form of Akt, the BV2 cells were plated into 24-well plates (5 × 10^4^ cells/mL); the next day, they were treated as described above. After 30 min of stimulation, the cells were harvested and washed with PBS. The cells were then fixed with 5% formalin for 10 min at RT and further permeabilized with ice-cold methanol for 20 min at 4 °C. Nonspecific staining was blocked with 3% bovine serum albumin in PBS for 30 min. Next, the cells were incubated with specific antibodies that recognize Akt phosphorylated at Ser473 (phospho-Akt[Ser473]; Santa Cruz Biotechnology) in blocking buffer for 45 min at 4 °C. Next, the cells were incubated with Alexa-Fluor-488-conjugated secondary antibodies for an additional 30 min at RT in the dark. Finally, the cells were washed twice with PBS and analyzed by flow cytometry (Attune NxT; Thermo Fisher Scientific). The data were analyzed using FlowJo software and are presented relative to the control cells.

#### Statistical Analysis

The data are presented as means ± SD and are representative of at least two independent experiments, each performed in at least duplicate. Statistical analysis was performed by one-way analysis of variance (ANOVA) followed by Tukey’s post hoc test using GraphPad Prism, version 6. A value of *p* < 0.05 was considered as the level of statistical significance.

## Results

### LPS and Poly(I:C) Induce Distinct Intensities of Proinflammatory Factor Release and Together Strengthen Microglia Activation

**Fig. 1:**
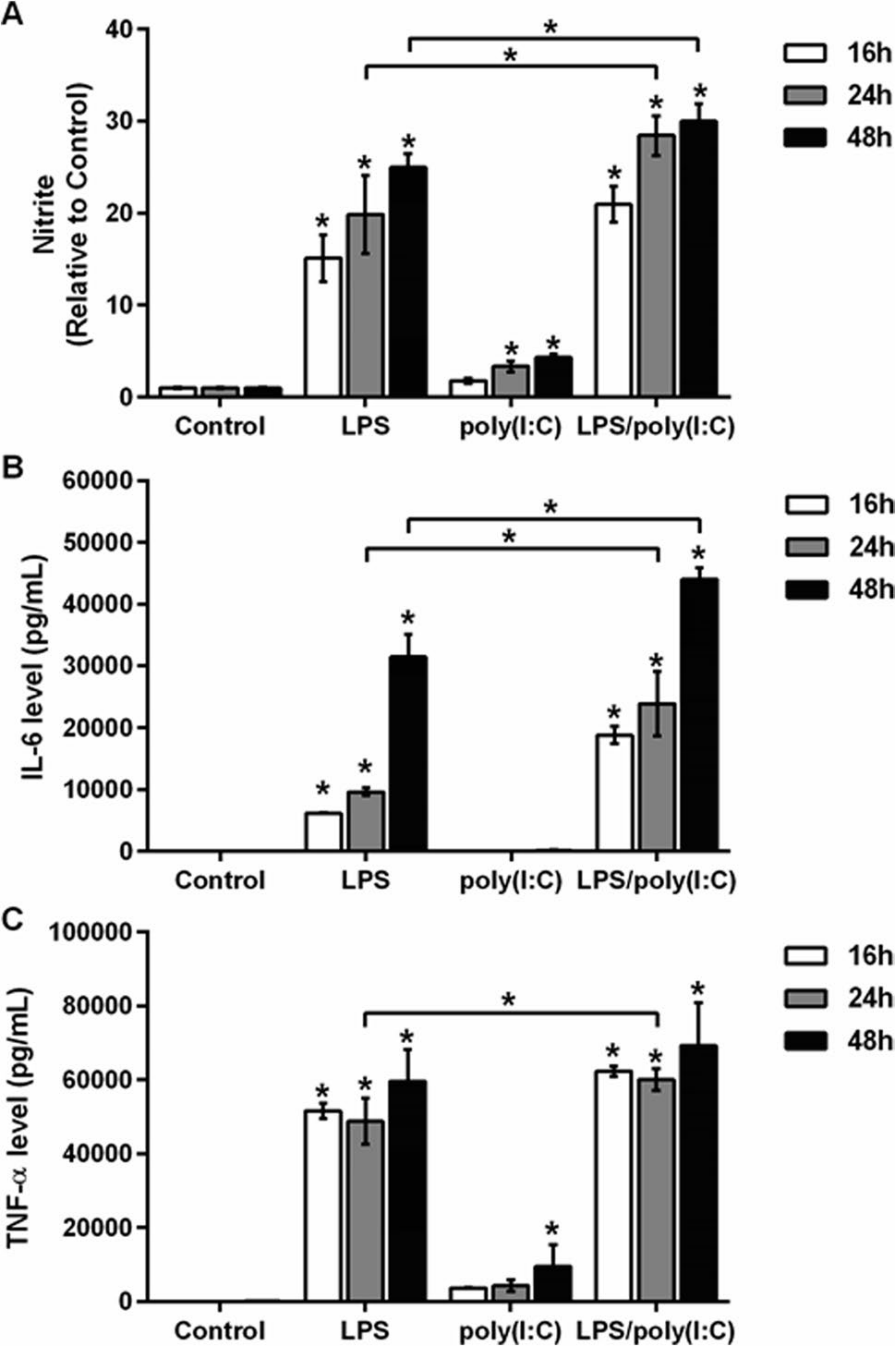
The effects of eimultaneous TLR3/4 activation on the intensity of microglia activation. BV2 cells were stimulated with 1 µg/mL LPS and/or 10 µg/mL poly(I:C) in serum-free medium for the times indicated. **A** Nitrite concentrations in the culture supernatants of the control and stimulated BV2 cells, determined using the Griess assay. Data are presented as means ± standard deviation of five independent experiments, each performed in duplicate. **B**, **C** Release of the cytokines IL-6 (**B**) and TNF-α (**C**) into the culture supernatants of the control and stimulated BV2 cells, determined using flow cytometry. Data are presented as means ± standard deviation of two independent experiments, each performed in duplicate. *p ˂ 0.05

The proinflammatory activity of microglia is characterized by the excessive release of NO and proinflammatory cytokines [50]; where the amounts of the released factors depend on the type and intensity of the inflammatory stimulus [3, 51]. LPS and poly(I:C) trigger similar inflammatory responses by activating TLR4 and TLR3, respectively [13]; however, the effects of simultaneous activation of these TLRs on microglia activation are not known. Therefore the release of proinflammatory factors into the culture medium by murine microglia BV2 cells was compared after individual and co-stimulation with LPS and poly(I:C). LPS alone significantly increased NO production in the medium of BV2 cells in a time-dependent manner (Fig. 1A). The increase in NO production was lower after stimulation with poly(I:C) alone, and only reached significance after 24 h and 48 h (Fig. 1A). However, when BV2 cells were simultaneously stimulated with both of these TLR4 and TLR3 ligands, a synergistic effect on NO production was observed, with significantly higher values than those of LPS and poly(I:C) alone after 24 h and 48 h of stimulation (Fig. 1A).

Increased expression of CD11b, the β-integrin marker of microglia, represents microglial activation during neurodegenerative inflammation and it has been shown that NO induces the expression of the cell surface microglia marker CD11b during microglia activation [52], therefore immunofluorescence staining of the activated BV2 cells was further performed. Compared to non-stimulated BV2 cells with low CD11b surface expression, stimulation with LPS alone or poly(I:C) alone increased CD11b surface expression. Co-activated (LPS plus poly(I:C)) BV2 cells exhibited an additional increase in CD11b expression (Supplementary Figure S1), which coincides with the results of elevated NO levels after simultaneously stimulated BV2 cells.

These data were supported by the measurements of the released proinflammatory cytokines in the culture medium of the BV2 cells stimulated in the same manner. As we reported previously [31], LPS significantly increased the cytokine IL-6 (Fig. 1B) and TNF-α (Fig. 1C) medium levels, with the most pronounced increases after 48 h. Conversely, poly(I:C) did not increase IL-6 levels, whereas it only significantly increased TNF-α levels after 48 h. Compared to LPS alone, the simultaneous stimulation with LPS and poly(I:C) significantly enhanced IL-6 levels after 24 h and 48 h (Fig. 1B), whereas TNF-α levels were increased to a lesser extent (Fig. 1C). These results indicate that the simultaneous activation of TLR4/TLR3 receptors strengthens microglia activation and results in more potent proinflammatory responses than those of the individual ligands alone.

### Co-stimulation with LPS and Poly(I:C) Affects Cathepsin X Expression and Activity Patterns more than Individual Stimulation

**Fig. 2:**
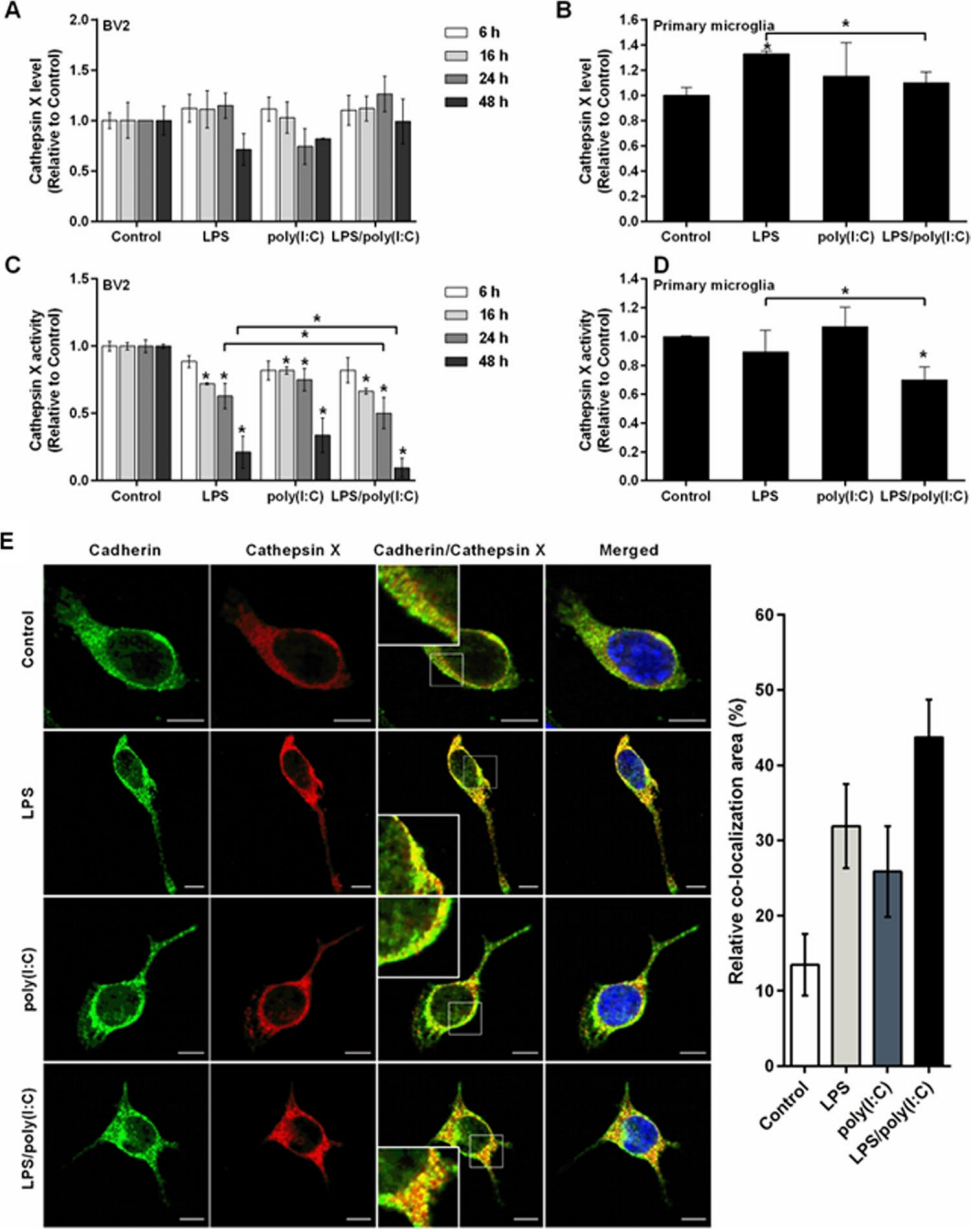
The effects of simultaneous TLR3/4 activation on cathepsin X expression and activity in microglia. **A**–**D** BV2 cells or primary microglia cultures were stimulated with 1 µg/mL LPS and/or 10 µg/mL poly(I:C) in serum-free medium for the times indicated (**A**, **C**) or for 24 h (**B**, **D**). **A**, **B** Cathepsin X protein levels in cell lysates of the control and stimulated cells were measured by ELISA, using a goat anti-cathepsin X capture antibody (AF934) and a mouse anti-cathepsin X detection antibody (3B10). Data are presented as means ± standard deviation of at least two independent experiments, each performed in duplicate. **C**, **D** Cathepsin X activity in cell lysates was determined using the cathepsin X-specific substrate Abz-Phe-Glu-Lys(Dnp)-OH. Data are presented as means ± standard deviation of at least two independent experiments, each performed in duplicate. *p ˂ 0.05. **E** Representative images of double immunofluorescence staining of cadherin (green) and cathepsin X (red) in unstimulated BV2 cells (control) and in BV2 cells stimulated with LPS and/or poly(I:C) for 24 h. Nuclei were counterstained with DAPI (blue). Right: quantification of the relative co-localization areas of cadherin and cathepsin X, presented as means ± standard deviation of pixels (cell numbers ≥ 10). Scale bars: 10 µm. *p ˂ 0.05

LPS-induced alterations in the activity and localization of cathepsin X in BV2 cells have been reported previously [31]. Therefore, we investigated here whether co-activation of these cells by both LPS and poly(I:C), which strengthens microglia activation, could further alter the expression and activity of cathepsin X. For this purpose, BV2 cells were stimulated with LPS and poly(I:C) alone and simultaneously for the time periods indicated in Fig. 2. Afterwards, the total cathepsin X protein levels and activities in cell lysates were determined using ELISA and enzymatic activity analysis, respectively. The cathepsin X protein levels after LPS stimulation were similar to those reported previously, with no significant changes except for a trend of decreased protein expression at 48 h that did not reach statistical significance (Fig. 2A). Similarly, poly(I:C) did not significantly affect cathepsin X expression levels, and only a trend of decreased protein expression at 24 h and 48 h was observed (Fig. 2A). Conversely, co-stimulation with both LPS and poly(I:C) increased protein levels at 24 h, but not significantly. Due to the noticeable difference at 24 h of co-stimulation, we further examined the cathepsin X expression pattern in activated primary microglia. Although no differences were observed compared to control cells of primary microglia, co-stimulation with both LPS and poly(I:C) significantly reduced the protein levels compared to LPS alone in primary microglia cultures (Fig. 2B). Additionally, we also determined the cathepsin X mRNA levels upon co-stimulation of microglial TLR4 and TLR3. Indeed, LPS and poly(I:C) alone or together increased cathepsin X mRNA levels at 24 h in BV2 cells, and co-stimulation revealed a trend of increased cathepsin X mRNA levels compared to each of the individual treatments (Supplementary Figure S2), which in turn correlates with the cathepsin X protein level at 24 h of co-stimulation of BV2 cells.

In contrast, LPS or poly(I:C) alone significantly decreased intracellular cathepsin X activity compared to the control BV2 cells, and these effects were even more pronounced after co-stimulation. Compared to LPS alone, co-stimulation further decreased cathepsin X activity in BV2 cells after 24 h and 48 h (Fig. 2C). Similarly, co-stimulation of primary microglia cultures for 24 h significantly decreased cathepsin X activity (Fig. 2D), which additionally confirms the effect of TLR3/4 receptor co-activation on decreased cathepsin X activity and suggests its secretion from activated microglia.

Due to these alterations in intracellular cathepsin X activity, the subcellular localization of cathepsin X was studied using the plasma membrane marker cadherin (Fig. 2E). In control BV2 cells, the co-localization of cathepsin X with cadherin was relatively weak, whereas after stimulation with LPS or poly(I:C) alone, the localization of cathepsin X at the plasma membrane was more apparent, although to a lesser extent for poly(I:C)-activated microglia. Co-stimulation strongly increased the localization of cathepsin X at the plasma membrane, as observed by increased co-localization of cathepsin X with cadherin (Fig. 2E). Additionally, the vesicular localization of cathepsin X in BV2 cells was analyzed using the lysosomal marker LAMP1. LPS or poly(I:C) stimulation alone for 24 h resulted in similar co-localization patterns of cathepsin X and LAMP1; the co-localization was less prominent than in the control cells. Co-stimulation further decreased the co-localization of cathepsin X and LAMP1 (Supplementary Figure S3), which further suggests peptidase secretion from activated microglia.

**Fig. 3:**
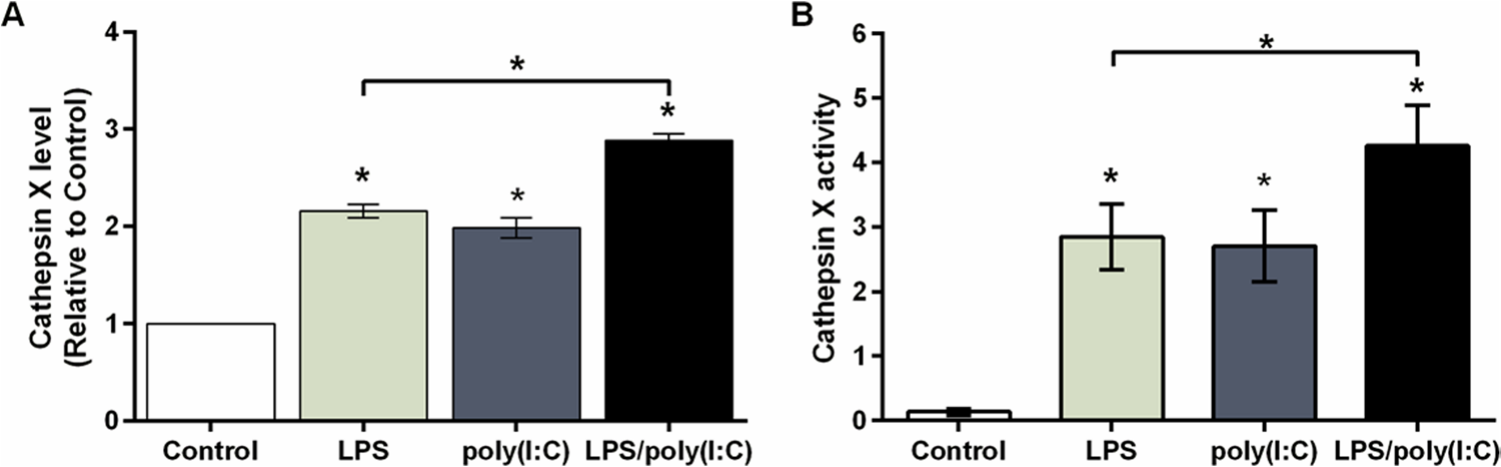
Simultaneous TLR3/4 activation affects cathepsin X release and localization in microglia. BV2 cells were stimulated with 1 µg/mL LPS and/or 10 µg/mL poly(I:C) for 24 h. **A** Cathepsin X protein levels in culture supernatants of control and stimulated BV2 cells were measured by ELISA, using a goat anti-cathepsin X capture antibody (AF934) and a mouse anti-cathepsin X detection antibody (3B10). Data are presented as means ± standard deviation of three independent experiments. **B** Cathepsin X activity in the culture supernatants was determined using the cathepsin X-specific substrate Abz-Phe-Glu-Lys(Dnp)-OH. Data are presented as means ± standard deviation of three independent experiments

Therefore, we further studied cathepsin X release into the culture medium after co-stimulation of TLR4 and TLR3. Cathepsin X protein levels and activity were thus determined in the culture medium obtained after stimulating BV2 cells with LPS and poly(I:C) alone or simultaneously. As already shown [31], LPS alone and poly(I:C) alone significantly increased extracellular cathepsin X protein levels after 24 h (Fig. 3A). Compared with these individual treatments, co-stimulation further increased cathepsin X secretion into the medium of BV2 cells after 24 h (Fig. 3A). A similar trend was also observed for stimulated and co-stimulated primary microglia cultures (Supplementary Figure S4). These results from the ELISA assays regarding the extracellular levels of cathepsin X were further supported by the extracellular activities of cathepsin X after 24 h. Cathepsin X activity was similarly increased in the culture medium of BV2 cells after individual LPS and poly(I:C) stimulation and further increased after co-stimulation (Fig. 3B), indicating on a significant role of secreted cathepsin X in the neurotoxic potential of strengthened microglia activation.

### Inhibiting Cathepsin X Activity Reduces LPS/Poly(I:C)-Enhanced Microglia Activation

**Fig. 4:**
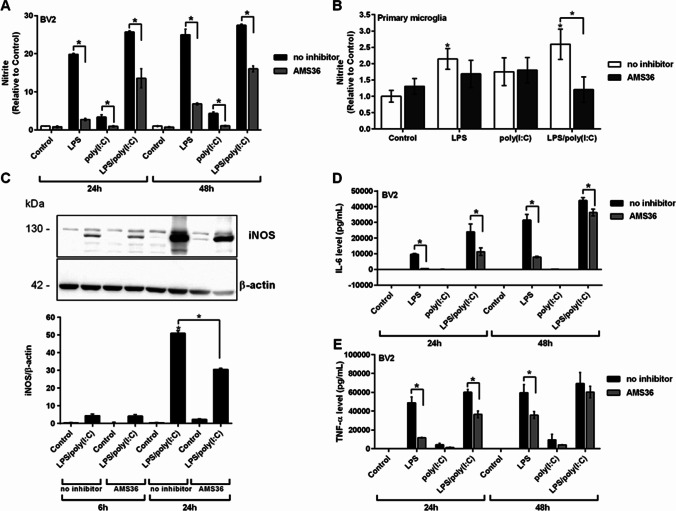
The effects of cathepsin X inhibition on the TLR3/4-mediated inflammatory response in microglia. BV2 cells or primary microglia cultures were pre-treated with cathepsin X inhibitor AMS36 (10 µM) for 1 h and then stimulated with 1 µg/mL LPS and/or 10 µg/mL poly(I:C) for the indicated times. **A** Nitrite concentrations in the culture supernatants of the control and stimulated BV2 cells, determined using the Griess assay. Data are presented as means ± standard deviation of five independent experiments, each performed in duplicate. **B** Nitrite concentrations in the culture supernatants of the control and stimulated primary microglia cultures for 24 h, determined using the Griess assay. Data are presented as means ± standard deviation of two independent experiments, each performed in duplicate. **C** Western blotting for iNOS expression in the BV2 cell lysates (*above*). The iNOS levels in the control and stimulated BV2 cells were quantified and normalized to the respective total β-actin, used as the loading control. Semi-quantitative densitometry analysis for the iNOS protein levels (*below*). Data are means of two independent experiments and are expressed relative to the control cells. **D**, **E** Release of the cytokines IL-6 (D) and TNF-α (E) into the culture supernatants of the control and stimulated BV2 cells, determined using flow cytometry. Data are means of two independent experiments, each performed in duplicate. *p ˂ 0.05

Inhibiting cathepsin X activity has been shown to reduce the production of proinflammatory mediators from LPS-activated BV2 cells [31]. To determine whether cathepsin X is also involved in LPS/poly(I:C)-enhanced microglia activation, BV2 cells were incubated with the cathepsin X inhibitor AMS36 prior to stimulation with LPS and/or poly(I:C). AMS36 alone did not affect NO levels in the culture medium. However, pre-treatment with AMS36 significantly decreased the NO levels in the culture medium of activated BV2 cells after 24 h and 48 h (Fig. 4A). A similar effect of AMS36 was observed in primary microglia cultures, as AMS36 inhibited the LPS- and poly(I:C)-induced increase in NO levels (Fig. 4B).

To confirm the role of cathepsin X, its expression in BV2 cells was reduced by transfection with specific siRNA oligonucleotides that target cathepsin X mRNA (siRNA-CatX) (Supplementary Figure S5A). siRNA-CatX significantly reduced NO production in co-stimulated cells compared to siRNA-Ctrl (Supplementary Figure S5B). Additionally, to exclude the involvement of other cysteine cathepsins, such as cathepsins B, L, and S, cells were pre-incubated with specific inhibitors against cathepsin B (CA-074; [53]), cathepsin L (CLIK-148; [54]), and cathepsin S (LHVS; [55]). Significant anti-inflammatory effects against LPS/poly(I:C)-induced microglia activation were observed for the cathepsin X inhibitor AMS36 (24 h). A smaller, but significant, effect was also observed for the cathepsin B inhibitor CA-074, but only after 24 h. None of the other cysteine cathepsin inhibitors exerted any effects (Supplementary Figure S6), indicating on the specific role of cathepsin X in the neurotoxic potential of activated microglia.

We then analyzed the effects of AMS36 on iNOS expression following LPS and poly(I:C) co-stimulation. In line with the changes in NO production, pre-treatment with AMS36 decreased LPS/poly(I:C)-induced iNOS protein levels after 24 h (Fig. 4C). Furthermore, the effect of AMS36 on the enhanced production of the proinflammatory cytokines IL-6 and TNF-α following LPS and poly(I:C) co-stimulation was analyzed. Indeed, AMS36 significantly decreased co-stimulation-enhanced IL-6 (Fig. 4D) and TNF-α (Fig. 4E) protein levels in the culture medium after 24 h. However, this protective effect of AMS36 was only significant after 24 h of co-stimulation. After 48 h, these inhibitory effects of AMS36 on the cytokine levels in the medium were less prominent compared to stimulation with LPS alone (Fig. 4D, E).

**Fig. 5:**
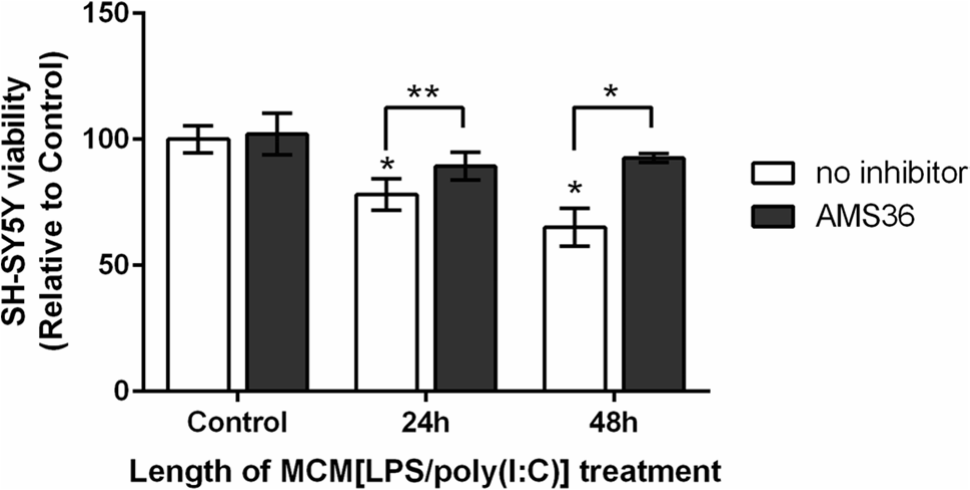
Neuroprotective effects of the cathepsin X inhibitor AMS36 on LPS/poly(I:C)-induced microglia-mediated neurotoxicity in SH-SY5Y cells. Microglia BV2 cells were pre-treated with 10 µM AMS36 for 1 h, followed by stimulation with 1 µg/mL LPS plus 10 µg/mL poly(I:C) for 24 h. Microglia culture supernatants were collected and incubated with neuronal SH-SY5Y cells for the times indicated. Neuronal cell viability was determined using the MTS assay. Data are presented as means ± standard deviation of two independent experiments, each performed in quadruplicate. *p ˂ 0.05; **p ˂ 0.01

Additionally, AMS36 was neuroprotective against neuronal cell death induced by microglia culture supernatants after LPS and poly(I:C) co-stimulation. When SH-SY5Y cells were treated with the culture medium from the BV2 cells co-stimulated with LPS and poly(I:C), they showed time-dependent decreases in cell viability. However, following pre-treatment of the BV2 cells with AMS36, the cytotoxic effects of the microglial culture medium on the SH-SY5Y cells were abrogated (Fig. 5), showing beneficial effect of microglia cathepsin X inhibition during microglia activation.

### Cathepsin X Inhibition Reduces LPS/Poly(I:C)-Enhanced Microglia Apoptosis

**Fig. 6:**
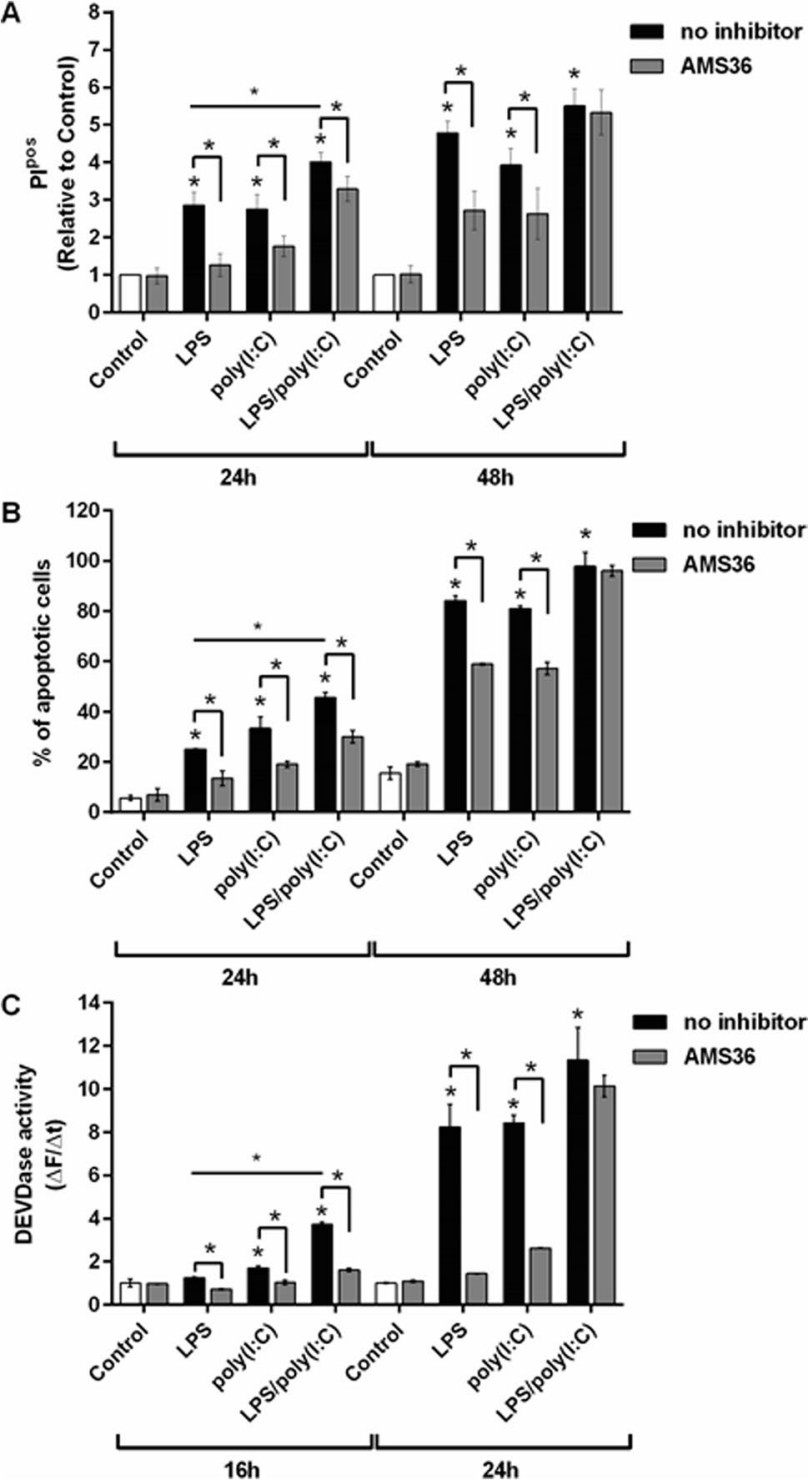
The effects of cathepsin X inhibition on TLR3/4-mediated apoptosis in microglia. BV2 cells were pre-treated with the cathepsin X inhibitor AMS36 (10 µM) for 1 h and then stimulated with 1 µg/mL LPS and/or 10 µg/mL poly(I:C) for the indicated times. **A** Cell death (apoptosis) of the control and stimulated BV2 cells was determined using propidium iodide (PI) and flow cytometry. Data are relative percentages of PI-positive (PI^pos^) cells normalized to the appropriate control, presented as means ± standard deviation of four independent experiments. **B** The proportions (%) of apoptotic cells determined using Annexin V and PI staining and flow cytometry. The quadrant threshold was set according to the control BV2 cells, and the quantitative analysis indicates the proportion (%) of the cells showing apoptosis (i.e., annexin-*V*^pos^ and PI^pos^). Data are presented as means ± standard deviation of two independent assays. **C** Caspase-3/7 activities in cell lysates of the control and stimulated BV2 cells, determined fluorometrically using the specific substrate for caspase 3/7, Ac-DEVD-AFC. Data are presented as rates of fluorescence changes (ΔF/Δt) with means ± standard deviation of four independent experiments, each performed in duplicate. *p ˂ 0.05

Microglia undergo apoptosis upon inflammatory activation in a manner similar to activation-induced lymphocyte death [56]. We have previously shown that inhibiting cathepsin X reduces LPS-induced microglia apoptosis in terms of caspase-3 activity and apoptotic cell staining [31]. Therefore, propidium iodide staining of microglia was also assessed here to compare this effect with that of cathepsin X inhibition with AMS36 after simultaneous TLR4 and TLR3 activation. AMS36 pre-treatment of BV2 cells almost completely prevented LPS-induced cell death (observed as propidium-iodide-positive cells) after 24 h. This protection was smaller after poly(I:C) treatment and was further reduced after co-stimulation after 24 h (Fig. 6A). However, after 48 h of stimulation, AMS36 still prevented cell death induced by the ligands individually but did not prevent cell death after co-stimulation. These effects were then further analyzed using a combination of annexin V and propidium iodide staining. These data were in agreement with the propidium iodide data; AMS36 did not prevent microglia apoptosis (observed as annexin-V-positive and propidium-iodide-positive staining) after 48 h of co-stimulation (Fig. 6B). These results were consistent with the caspase-3 activity measurements; AMS36 pre-treatment prevented cell apoptosis due to LPS/poly(I:C) co-stimulation after 24 h, but this protective effect was lost after 48 h of co-stimulation (Fig. 6C). Therefore, these data indicate an active role of cathepsin X in at least the early stages of activation-induced microglia apoptosis.

To further investigate the effects of cathepsin X inhibition, we considered the additional receptor-interacting protein kinase-3-mediated programmed cell death pathway in TLR-activated microglia that was recently identified and termed necroptosis [57]. The zVAD-fmk inhibitor reduced intracellular cathepsin X activity in BV2 cells after 24 h of LPS/poly(I:C) co-stimulation but not after 48 h (Supplementary Figure S7).

### PI3K Inhibition Reduces the LPS/Poly(I:C)-Enhanced Proinflammatory Response and Affects Cathepsin X Activity Regulation

**Fig. 7:**
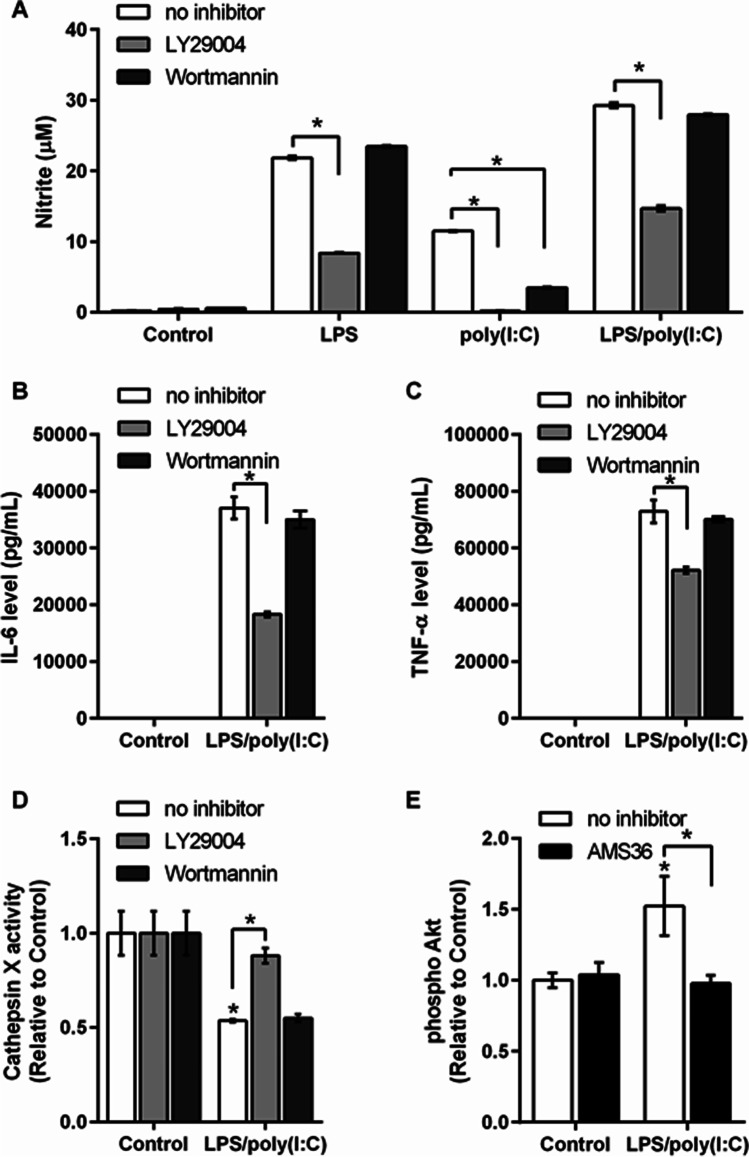
LY29004 modulates the LPS/poly(I:C)-induced proinflammatory response and cathepsin X activity. **A**–**D** BV2 cells were pre-treated with 10 µM LY29004 or 1 µM wortmannin for 1 h and then stimulated with 1 µg/mL LPS and/or 10 µg/mL poly(I:C) for 24 h. **A** Nitrite concentrations in the culture supernatants of the control and stimulated BV2 cells, determined using the Griess assay. Data are presented as means ± standard deviation of two independent experiments, each performed in duplicate. **B**, **C** Levels of the cytokines IL-6 (**B**) and TNF-α (**C**) in the culture supernatants of the control and LPS/poly(I:C)-stimulated BV2 cells, determined using flow cytometry. Data are means of two independent experiments, each performed in duplicate. **D** Cathepsin X activity in cell lysates was determined using the cathepsin X-specific substrate, Abz-Phe-Glu-Lys(Dnp)-OH. Data are presented as means ± standard deviation of two independent experiments, each performed in duplicate. **E** Analysis of Akt activation. BV2 cells were pre-treated with the cathepsin X inhibitor AMS36 (10 µM) for 1 h, followed by 1 µg/mL LPS and 10 µg/mL poly(I:C) stimulation for 30 min. Flow cytometry analysis of Akt activation was performed using a specific antibody against phosphorylated Ser473 of Akt. Data are means of two independent experiments, each performed in duplicate. *p ˂ 0.05

The common signal transduction pathway activated through TLR4 and TLR3 includes PI3K/Akt, whereby NF-κB activation further downstream has an important role in microglia activation and neuroinflammation [58]. Therefore, we investigated the effects of inhibitors of the PI3K pathway on this pronounced LPS/poly(I:C)-induced proinflammatory response in relation to cathepsin X regulation. BV2 cells were pre-treated with the reversible and irreversible PI3K inhibitors LY294002 and wortmannin, respectively, followed by stimulation with LPS and poly(I:C) individually and simultaneously. Then NO production was assessed. Following pre-treatment with LY294002, NO production was reduced after LPS treatment, completely blocked after poly(I:C) treatment, and reduced by their combination after 24 h (Fig. 7A). In contrast, pre-treatment with wortmannin exerted no effects on LPS- and LPS/poly(I:C)-induced NO production; however, wortmannin reduced NO levels after stimulation with poly(I:C) alone (Fig. 7A). Similarly, the LPS/poly(I:C)-induced levels of IL-6 and TNF-α in the culture medium of BV2 cells co-activated for 24 h were also reduced by LY294002, whereas wortmannin exerted no effects (Fig. 7B, C).

Furthermore, the effects of the two PI3K inhibitors on intracellular cathepsin X activity were evaluated. Pre-treatment of BV2 cells with LY294002 reversed the reduced cathepsin X activity as a response to LPS/poly(I:C) co-stimulation (Fig. 7D). In contrast, wortmannin had no effect on intracellular cathepsin X activity (Fig. 7D).

Finally, we investigated whether cathepsin X activity affects the PI3K/Akt pathway. We quantified the phosphorylation levels of the downstream target of PI3K, Akt, in its active form [59], in BV2 cells. The phosphorylated Akt levels were significantly increased after 30 min of LPS/poly(I:C) co-stimulation; however, this phosphorylation of Akt was completely blocked by pre-treatment with AMS36, i.e., by cathepsin X inhibition (Fig. 7E). These results indicate that cathepsin X is involved in regulating the PI3K/Akt signaling pathway that is activated by TLR4 and TLR3 ligands, which trigger microglia activation and inflammatory responses.

## Discussion

Microglia are immune-like cells in the brain that play an important role in inflammatory responses in the CNS. Although there is a great body of evidence that shows that TLR signaling has beneficial effects in the CNS, it has become evident that TLR-induced activation of microglia and the release of proinflammatory molecules are responsible for neurotoxic processes in various CNS diseases [3]. Thus, inhibiting microglia activation represents a promising strategy in treating inflammation-associated neurodegenerative disorders.

We have previously shown that cathepsin X is a key mediator in neurodegeneration mediated by microglia activation and that cathepsin X inhibition reduces LPS-induced microglia activation [31]. In the current study, we first analyzed the effect of simultaneous TLR4 and TLR3 activation with LPS and poly(I:C) on microglia-mediated proinflammatory responses. Second, we further examined the involvement of cathepsin X in enhanced microglia activation caused by LPS and poly(I:C) co-stimulation.

TLR activation can contribute to both infectious and noninfectious CNS diseases [60]. Approaches to unravel the influence of TLR activation in the CNS that are based on cell models have mainly used single ligands that are highly specific for the activation of their respective TLR. However, microglia constitutively express mRNA of most of the TLRs identified to date [61], and TLR expression appears to be regulated by the engagement of its ligands [62]. Therefore, it can be reasonably assumed that more than one TLR is involved in physiological and pathological processes within the CNS, and that the ligands that activate the various TLRs are likely present as a mixture at any one point in time. Indeed, Rosenberg et al. demonstrated the effect of simultaneous TLR activation on neuroinflammation and neurodegeneration [41]. Co-stimulation of microglial TLR4 and TLR2, TLR4 and TLR9, and TLR2 and TLR9 by their respective specific ligands resulted in increased inflammatory responses compared to activating their respective individual TLRs. However, not all these TLRs act in synergy, as they showed that combined stimulation of TLR3 and TLR4 did not affect the TNF-α response [41]. In the present study, however, we have shown that co-stimulation of TLR4 and TLR3 by their respective ligands LPS and poly(I:C) results in increased secretion of the inflammatory molecule NO from microglia, unlike the activation of the respective individual TLRs. Additionally, greater secretion of the proinflammatory molecules IL-6 and TNF-α was induced by LPS and poly(I:C) combined, as compared with TLR3 activation alone, which induced only minimal increases in NO, IL-6, and TNF-α levels. The differences between the effects of LPS and poly(I:C) on proinflammatory responses are in line with other reports, which have emphasized that these responses exhibit distinct intensity patterns [13, 14]. Here, we have further demonstrated that co-stimulation of microglia with LPS and poly(I:C) further increases IL-6 secretion after 24 h and 48 h. This effect was observed to a lesser extent for TNF-α secretion.

The synergy of TLR3 and TLR4 activation on cathepsin X expression and localization patterns in microglia was also shown in this study. To date, no reports have assessed the effects of simultaneously activating different TLRs on the regulation of cysteine cathepsins. Stimulation of microglia with LPS alone has been shown to increase the expression, release, and activity of certain cathepsins, such as cathepsin B [23, 63], cathepsin C [29], cathepsin H [28], and cathepsin X [26, 31, 34]. In BV2 cells, we previously demonstrated that LPS increases cathepsin X levels and activity in the culture medium and decreases the intracellular activity of cathepsin X without altering the intracellular levels [31]. In the present study, similar intracellular cathepsin X protein levels were observed in BV2 cells after stimulation with either poly(I:C) or LPS alone. LPS/poly(I:C) co-stimulation did not affect cathepsin X protein expression but increased cathepsin X mRNA levels after 24 h. Individual stimulation with LPS and poly(I:C) reduced intracellular cathepsin X activity in a time-dependent manner. Simultaneously activating both TLR4 and TLR3 by these respective ligands for 24 h resulted in synergistic effects, as intracellular cathepsin X activity was additionally reduced compared to the effects of the individual ligands. The same effects were observed in the primary microglia cultures. However, the intracellular cathepsin X protein levels were not in agreement with the intracellular cathepsin X activity after TLR stimulation. The latter is in line with a regulation of cysteine cathepsins during TLR responses in macrophages with increased activities of cathepsins B, L, and S after the individual activation of TLR2, TLR3, and TLR4, whereas the levels of the transcripts encoding the cathepsins were unchanged [64].

Conversely, in the present study, we show that the mRNA cathepsin X levels are increased and that the cathepsin X protein levels correlate well with cathepsin X activity in culture supernatants (i.e., secreted cathepsin X). Both the extracellular protein and activity levels of cathepsin X were increased after individual stimulation with LPS or poly(I:C). Co-stimulation with LPS and poly(I:C) further increased cathepsin X protein secretion. Likewise, the proteolytic activity of cathepsin X in the culture supernatant of the co-activated BV2 cells was more pronounced than the activities stimulated by the individual ligands. Similarly, inducible effect on release and enzymatic activity of cathepsin X in primary microglia has been observed; however, it is modest compared to the response of the BV2 microglia. Similarly, other studies also showed lower protein levels of some cysteine cathepsins, as such cathepsins C and H, in the culture medium of primary microglia than of BV2 cells following LPS stimulation, suggesting that the difference reflects biological characteristics of two types of cells [28, 29]. Additionally, subcellular localization of cathepsin X at the plasma membrane following LPS/poly(I:C) co-stimulation was more prominent than that following stimulation by the individual ligands. In contrast, the intensity of the cathepsin X signal in the lysosomal vesicles in the co-stimulated BV2 cells was decreased. This decrease is in line with the increased release of cathepsin X from activated microglia as a response to the synergy of TLR3 and TLR4 activation. These findings correlate with other studies as it has been already shown that cathepsins are either localized intracellularly (within lysosomes) or extracellularly [25] and are differentially expressed in microglia in response to pro-inflammatory stimuli [65, 66]. Factors released by microglia can kill neurons directly by promoting neuronal self-destruction or indirectly by promoting non-neuronal cells to produce other factors that induce neuronal death [66]. In vitro exposure to inflammatory stimuli (e.g., LPS) increases the levels of certain cysteine cathepsins in culture supernatants of the microglia cell line BV2 [26, 67]. For cathepsin B, it has been shown that upon pro-inflammatory stimulation, it is secreted from activated microglia, which has been shown to be a major causative factor of microglia-induced neuronal apoptosis [23]. Similarly, cathepsin X is strongly associated with microglia polarization towards the neurotoxic phenotype. The proteolytic activity of cathepsin X in culture supernatants of activated microglial cells can be evoked by LPS stimulation and can lead to neurotoxicity mediated by microglia activation [26, 31]. First, cathepsin X promotes 6-hydroxydopamine-induced apoptosis of PC12 and SH-SY5Y cells through caspase cascade and NF-kB activation. [44]. Second, cathepsin X is a modulator of the mitogen-activated protein kinase signaling pathway in activated microglia [31]. This pathway plays a critical role in the production of cytokines and mediators associated with the pathogenesis of inflammation [68] and inhibition of cathepsin X proteolytic activity by AMS36 in LPS-activated BV2 cells markedly blocked LPS-induced p38 and c-Jun N-terminal kinase activation and reduced LPS-induced phosphorylation of extracellular signal-regulated kinases 1 and 2, suppressing the increased cytokine release from activated microglia [31]. Therefore, increased expression and secretion of cathepsin X support roles of neurotoxic phenotype of activated microglia as has been also discussed for various cathepsins (reviewed in [69]).

Activated microglia should initially protect neurons during induced degeneration. However, the inflammatory products derived from microglia have been implicated in the neuronal destruction that is commonly observed in various neurodegenerative diseases [70]. Activated microglia also synthesize and secrete cathepsins, particularly during the early stage of inflammation, triggering signaling pathways in a pathological cascade that aggravates neuroinflammation [26–28, 31, 71]. Additionally, the autocrine and/or paracrine features of the secreted proinflammatory molecules might also contribute to enhanced inflammatory responses [22, 72]. Therefore, better regulation of microglia activation and inactivation of cathepsins might represent a key strategy in treating inflammation-associated neurodegeneration. Inhibiting cathepsin X has been shown to successfully reduce the increased release of NO and other proinflammatory molecules from LPS-activated microglia, reducing microglia-mediated neurotoxicity [31]. Here, inhibiting cathepsin X with AMS36 also reduced the enhanced release of NO from microglia resulting from the co-stimulation of TLR4 and TLR3 by their respective ligands. However, except for small (but significant) effects of the cathepsin B inhibitor CA-074, the cathepsin L and S inhibitors CLIK-148 and LHVS, respectively, did not exert anti-inflammatory effects against this increased microglia activation. Increased NO is due to enhanced iNOS expression [73], which occurs primarily in microglia in response to extracellular stimuli, including LPS, IL-1β, IFN-γ, and TNF-α [74, 75]. Indeed, here we observed increased iNOS expression after LPS and poly(I:C) co-stimulation compared to individual ligand stimulation; these increased iNOS levels were reduced after AMS36 pre-treatment. Likewise, inhibiting cathepsin X with AMS36 reduced the LPS/poly(I:C)-induced increase in IL-6 and TNF-α levels in the culture supernatants, with a greater effect after 24 h than after 48 h of co-stimulation. Furthermore, inhibiting cathepsin X not only suppressed microglia activation, and consequently the inflammatory response through NO, but also affected the viability of microglia. The synergy of this TLR4 and TLR3 co-activation of microglia resulted in increased cell death compared to the individual TLR ligands. Thus, inhibiting cathepsin X protected the microglia against LPS/poly(I:C)-induced cell death. However, this protection was only observed after 24 h of co-stimulation and not after 48 h. This correlates with the AMS36-induced suppression of proinflammatory responses observed after 24 h.

When microglia are activated by inflammatory stimuli, they can undergo apoptosis as a possible autoregulatory mechanism for their own activation state. Indeed, the NO produced by such activated microglia is the primary cytotoxic mediator in this process. Inflammatory stimuli like LPS play multiple roles in microglia apoptosis. LPS not only induces cytotoxic NO production but also initiates the NO-independent apoptotic pathway through stimulation of the caspase cascade [56]. The synergistic effects of TLR4 and TLR3 activation were also observed for this increased apoptosis. Co-stimulation with LPS and poly(I:C) increased apoptosis of BV2 cells more than individual ligands. Cysteine cathepsins have been suggested to interfere with the apoptotic cascade in microglia by activating caspases [76]. Indeed, pre-treatment with the cathepsin X inhibitor AMS36 decreased the level of apoptosis after 24 h of co-stimulation, but this protection against apoptosis was lost after 48 h. These effects on apoptosis were therefore also examined in terms of the caspase cascade. The NO-independent cytotoxic mechanism involves up-regulation of caspase-11, which is autoactivated in microglia and triggers an activation cascade of downstream caspases, ultimately leading to cell apoptosis [56]. Caspase-3 is the final executor of this caspase-dependent apoptotic damage, and its activity was significantly increased in microglia upon individual stimulation with LPS and poly(I:C). This increased caspase-3 activity was more pronounced after LPS/poly(I:C) co-stimulation. However, the reduced caspase-3 activity due to cathepsin X inhibition was only observed after 16 h, and not afterwards.

However, the effects of cathepsin X inhibition, mediated through reduced proinflammatory factors, are not congruent with the protective role of cathepsin X inhibition against microglia cell death that arises from the simultaneous activation of TLR4 and TLR3. Thus, cathepsin X might be involved in RIP3-mediated programmed necrosis. This additional RIP3-mediated programmed cell death pathway in TLR-activated microglia was recently identified and is also termed necroptosis [57]. It was shown that microglia activated by TLR4 or TLR3 ligands (such as LPS or poly(I:C), respectively) undergo RIP1/RIP3-dependent necroptosis when exposed to the pan-caspase inhibitor zVAD-fmk [57]. In the present study, exposing BV2 cells to zVAD-fmk reduced the intracellular cathepsin X activity observed after 24 h of LPS/poly(I:C) co-stimulation, but not after 48 h. This thus defines a time-dependent effect of cathepsin X inhibition on microglia apoptosis.

The intensity of the inflammatory response elicited by simultaneous TLR3 and TLR4 activation appears to result from the common regulatory adaptor protein and/or signaling pathway. There is evidence that after TLR3 or TLR4 activation, Akt is activated downstream via the TRIF pathway of TLR signaling [77]. Akt is a serine/threonine protein kinase that is activated during cytokine stimulation [78]. In addition, Akt might also regulate the activity of transcription factors, including NF-κB [79], and LPS-induced NF-κB activation in microglia is directly regulated by phosphorylation of Akt through the PI3K/Akt pathway [80]. For this reason, we investigated the PI3K/Akt pathway as a possible underlying mechanism of the synergy of TLR4 and TLR3 co-activation.

Treatment with the reversible PI3K inhibitor LY294002 before LPS and poly(I:C) individual stimulation or co-stimulation resulted in decreased NO levels in the culture supernatants of BV2 cells. In contrast, the alternative PI3K inhibitor, wortmannin, did not reduce the LPS- or co-stimulation-induced NO levels but did inhibit the NO levels induced by poly(I:C) alone. These observations are in line with other reports that have shown differential effects of LY294002 and wortmannin on proinflammatory factors resulting from LPS stimulation [58, 81]. Additionally, the enhanced levels of the cytokines IL-6 and TNF-α following simultaneous TLR3 and TLR4 activation were reduced by LY294002 but not by wortmannin, as observed in other studies [58, 82–84]. We noted that LY294002, but not wortmannin, modulated the activity of cathepsin X, as LY294002 reversed the reduced cathepsin X activity following LPS/poly(I:C) co-stimulation. Tsai et al. also showed differential effects of LY294002 and wortmannin on inducible nitric oxide synthase expression in glomerular mesangial cells. LY294002 showed a more-significant inhibitory effect on LPS/IFN-γ-induced NO production and exhibited a more-significant inhibitory effect on NF-κB luciferase activities than wortmannin in LPS/IFN-γ-stimulated MES-13 cells. Moreover, LY294002, but not wortmannin, accelerated iNOS protein degradation and reduced the iNOS dimer/monomer ratio in MES-13 cells, which indicates that the effects of LY294002 on inhibiting NF-κB activation and accelerating iNOS protein degradation are through a mechanism independent of PI3K [81]. Additionally, Zhao et al. showed that LY294002 inhibited LPS- and poly(I:C)-induced interferon β transcription and secretion from macrophages. In contrast, wortmannin could not inhibit interferon β production. Furthermore, interferon regulatory factor 3 transcriptional activation and binding to interferon β promoter were found to be inhibited by LY294002. Therefore, findings demonstrate LY294002 negatively regulates LPS- and poly(I:C)-induced interferon β production through inhibition of interferon regulatory factor 3 activation in a PI3K-independent manner [58].

Nevertheless, in the present study cathepsin X inhibition decreased the activation of the PI3K/Akt signaling pathway resulting from LPS/poly(I:C) co-stimulation. Kim and Li proposed that microglia activated through TLRs can undergo apoptotic self-elimination or RIP3-mediated programmed necrosis when sensitized by the pan-caspase inhibitor zVAD-fmk [57]. Recently, by targeting RIP3, Hu et al. reported that PI3K is essential for TNF-induced necroptosis [85]. The data obtained in the present study demonstrate that the PI3K/Akt pathway is important for the inflammatory activation of microglia, in which cathepsin X might play an important role. However, further investigations are needed to unambiguously define the involvement of cathepsin X in necroptosis in activated microglia.

## Conclusions

In the present study, we tested the hypothesis that co-stimulation of microglia with TLR3 and TLR4 ligands can provide synergism in the microglia inflammatory response. Indeed, our data show that LPS and poly(I:C) individually induce distinct intensities of proinflammatory factor release, while together (i.e., co-stimulation), they strengthen the activation of microglia. More importantly, we provide evidence that cathepsin X is involved in the increased activation of microglia and subsequent neuroinflammation, as the cathepsin X inhibitor AMS36 protected neuronal cells against microglia-mediated neurotoxicity. These data help to clarify the role of these different TLRs in microglia activation and pave the way towards new therapeutic strategies. Given the pathogenic role of cathepsin X in neuroinflammation, cathepsin X inhibitors could be helpful in alleviating the neurodegenerative processes associated with microglia-mediated inflammation.

## Supplementary Information

Below is the link to the electronic supplementary material.

Supplementary file1 (DOC 2720 KB)

## Data Availability

All data generated or analyzed during this study are included in this published article (and its supplementary information files). The datasets used and/or analyzed during the current study are also available from the corresponding author.

## Abbreviations

CNS: Central nervous system
IL: Interleukin
iNOS: Inducible nitric oxide synthase
LHVS: Leucine-homophenylalanine-vinyl phenyl sulfone
LPS: Lipopolysaccharide
NF-κB: Nuclear factor kappa B
NO: Nitric oxide
PAMPs: Pathogen-associated molecule patterns
PBS: Phosphate-buffered saline
PI3K: Phosphoinositide 3 kinase
Poly(I:C): Polyinosinic-polycytidylic acid
RT: Room temperature
siRNA: Small interfering RNA
TLR: Toll-like receptor
TNF: Tumor necrosis factor

## References

[1] Kawai T, Akira S (2007). Antiviral signaling through pattern recognition receptors. J Biochem.

[2] Carpentier PA, Duncan DS, Miller SD (2008). Glial toll-like receptor signaling in central nervous system infection and autoimmunity. Brain Behav Immun.

[3] Lehnardt S (2010). Innate immunity and neuroinflammation in the CNS: the role of microglia in toll-like receptor-mediated neuronal injury. Glia.

[4] Yang I (2010). The role of microglia in central nervous system immunity and glioma immunology. J Clin Neurosci.

[5] Hanisch UK, Kettenmann H (2007). Microglia: active sensor and versatile effector cells in the normal and pathologic brain. Nat Neurosci.

[6] Perry VH, Nicoll JAR, Holmes C (2010). Microglia in neurodegenerative disease. Nat Rev Neurol.

[7] Gomez-Nicola D, Perry VH (2015). Microglial dynamics and role in the healthy and diseased brain: a paradigm of functional plasticity. Neuroscientist.

[8] Aravalli RN, Peterson PK, Lokensgard JR (2007). Toll-like receptors in defense and damage of the central nervous system. J Neuroimmune Pharmacol.

[9] Medzhitov R, Janeway CA (2002). Decoding the patterns of self and nonself by the innate immune system. Science.

[10] Underhill DM (2007). Collaboration between the innate immune receptors dectin-1, TLRs, and Nods. Immunol Rev.

[11] Figdor CG, van Kooyk Y, Adema GJ (2002). C-type lectin receptors on dendritic cells and Langerhans cells. Nat Rev Immunol.

[12] Kawai T, Akira S (2010). The role of pattern-recognition receptors in innate immunity: update on Toll-like receptors. Nat Immunol.

[13] Lee HJ (2007). Differences between lipopolysaccharide and double-stranded RNA in innate immune responses of BV2 microglial cells. Int J Neurosci.

[14] Reimer T (2008). poly(I:C) and LPS induce distinct IRF3 and NF-kappaB signaling during type-I IFN and TNF responses in human macrophages. J Leukoc Biol.

[15] Poltorak A (1998). Defective LPS signaling in C3H/HeJ and C57BL/10ScCr mice: Mutations in Tlr4 gene. Science.

[16] Visintin A (2003). Lysines 128 and 132 enable lipopolysaccharide binding to MD-2, leading to Toll-like receptor-4 aggregation and signal transduction. J Biol Chem.

[17] Jiang ZF (2005). CD14 is required for MyD88-independent LPS signaling. Nat Immunol.

[18] Weber F (2006). Double-stranded RNA is produced by positive-strand RNA viruses and DNA viruses but not in detectable amounts by negative-strand RNA viruses. J Virol.

[19] Alexopoulou L (2001). Recognition of double-stranded RNA and activation of NF-kappaB by Toll-like receptor 3. Nature.

[20] Nishiya T (2005). TLR3 and TLR7 are targeted to the same intracellular compartments by distinct regulatory elements. J Biol Chem.

[21] Faure E (2000). Bacterial lipopolysaccharide activates NF-kappa B through Toll-like receptor 4 (TLR-4) in cultured human dermal endothelial cells - Differential expression of TLR-4 and TLR-2 in endothelial cells. J Biol Chem.

[22] Kettenmann H (2011). Physiology of microglia. Physiol Rev.

[23] Kingham PJ, Pocock JM (2001). Microglial secreted cathepsin B induces neuronal apoptosis. J Neurochem.

[24] Nakanishi H (2003). Microglial functions and proteases. Mol Neurobiol.

[25] Nakanishi H (2003). Neuronal and microglial cathepsins in aging and age-related diseases. Ageing Res Rev.

[26] Wendt W (2009). Intra- versus extracellular effects of microglia-derived cysteine proteases in a conditioned medium transfer model. J Neurochem.

[27] Clark AK, Malcangio M (2012). Microglial signalling mechanisms: Cathepsin S and Fractalkine. Exp Neurol.

[28] Fan K, et al (2015) The induction of neuronal death by up-regulated microglial cathepsin H in LPS-induced neuroinflammation. J Neuroinflammation 12 10.1186/s12974-015-0268-x PMC4379721 25889123

[29] Fan K, et al (2012) Up-regulation of microglial cathepsin C expression and activity in lipopolysaccharide-induced neuroinflammation. J Neuroinflammation 9 10.1186/1742-2094-9-96 PMC3410810 22607609

[30] Hafner A (2013). Neuroprotective role of gamma-enolase in microglia in a mouse model of Alzheimer's disease is regulated by cathepsin X. Aging Cell.

[31] Pislar A (2017). Inhibition of cathepsin X reduces the strength of microglial-mediated neuroinflammation. Neuropharmacology.

[32] Qi R, Singh D, Kao CC (2012). Proteolytic processing regulates Toll-like receptor 3 stability and endosomal localization. J Biol Chem.

[33] Stichel CC, Luebbert H (2007). Inflammatory processes in the aging mouse brain: Participation of dendritic cells and T-cells. Neurobiol Aging.

[34] Pislar A et al (2020) Neuroinflammation-induced upregulation of glial cathepsin X expression and activity in vivo. Front Mol Neurosci 13 575453 10.3389/fnmol.2020.575453 PMC7714997 33328882

[35] Kos J (2005). Carboxypeptidases cathepsins X and B display distinct protein profile in human cells and tissues. Exp Cell Res.

[36] Wendt W (2007). Differential expression of cathepsin X in aging and pathological central nervous system of mice. Exp Neurol.

[37] Tseveleki V (2010). Comparative gene expression analysis in mouse models for multiple sclerosis, Alzheimer's disease and stroke for identifying commonly regulated and disease-specific gene changes. Genomics.

[38] Glanzer JG (2007). Genomic and proteomic microglial profiling: pathways for neuroprotective inflammatory responses following nerve fragment clearance and activation. J Neurochem.

[39] Greco TM (2010). Quantitative mass spectrometry-based proteomics reveals the dynamic range of primary mouse astrocyte protein secretion. J Proteome Res.

[40] Allan ERO (2017). A role for cathepsin Z in neuroinflammation provides mechanistic support for an epigenetic risk factor in multiple sclerosis. J Neuroinflammation.

[41] Rosenberger K (2014). The impact of single and pairwise Toll-like receptor activation on neuroinflammation and neurodegeneration. J Neuroinflammation.

[42] Chen X et al (2013) Isolation, purification, and culture of primary murine microglia cells. Bio-protocol 3:e314

[43] Sadaghiani AM (2007). Design, synthesis, and evaluation of in vivo potency and selectivity of epoxysuccinyl-based inhibitors of papain-family cysteine proteases. Chem Biol.

[44] Pislar AH (2014). Cathepsin X promotes 6-hydroxydopamine-induced apoptosis of PC12 and SH-SY5Y cells. Neuropharmacology.

[45] Hafner A, Obermajer N, Kos J (2010). gamma-1-syntrophin mediates trafficking of gamma-enolase towards the plasma membrane and enhances its neurotrophic activity. Neurosignals.

[46] Bryan NS, Grisham MB (2007). Methods to detect nitric oxide and its metabolites in biological samples. Free Radic Biol Med.

[47] Andersen CL, Jensen JL, Orntoft TF (2004). Normalization of real-time quantitative reverse transcription-PCR data: a model-based variance estimation approach to identify genes suited for normalization, applied to bladder and colon cancer data sets. Cancer Res.

[48] Hafner A, Obermajer N, Kos J (2012). gamma-Enolase C-terminal peptide promotes cell survival and neurite outgrowth by activation of the PI3K/Akt and MAPK/ERK signalling pathways. Biochem J.

[49] Murn J, Urleb U, Mlinaric-Rascan I (2004). Internucleosomal DNA cleavage in apoptotic WEHI 231 cells is mediated by a chymotrypsin-like protease. Genes Cells.

[50] Block ML, Hong JS (2005). Microglia and inflammation-mediated neurodegeneration: multiple triggers with a common mechanism. Prog Neurobiol.

[51] Li L (2007). The function of microglia, either neuroprotection or neurotoxicity, is determined by the equilibrium among factors released from activated microglia in vitro. Brain Res.

[52] Roy A (2006). Up-regulation of microglial CD11b expression by nitric oxide. J Biol Chem.

[53] Montaser M, Lalmanach G, Mach L (2002). CA-074, but not its methyl ester CA-074Me, is a selective inhibitor of cathepsin B within living cells. Biol Chem.

[54] Katunuma N (2011). Structure-based development of specific inhibitors for individual cathepsins and their medical applications. Proc Jpn Acad Ser B Phys Biol Sci.

[55] Xu J et al (2013) Inhibition of cathepsin S produces neuroprotective effects after traumatic brain injury in mice. Mediators Inflamm 2013 187873 10.1155/2013/187873 PMC3824312 24282339

[56] Lee P (2001). NO as an autocrine mediator in the apoptosis of activated microglial cells: correlation between activation and apoptosis of microglial cells. Brain Res.

[57] Kim SJ, Li J (2013) Caspase blockade induces RIP3-mediated programmed necrosis in Toll-like receptor-activated microglia. Cell Death Dis 4 e716 10.1038/cddis.2013.238 PMC3730412 23846218

[58] Zhao W (2012). LY294002 inhibits TLR3/4-mediated IFN-beta production via inhibition of IRF3 activation with a PI3K-independent mechanism. FEBS Lett.

[59] Cianciulli A et al (2020) Microglia mediated neuroinflammation: focus on PI3K modulation. Biomolecules 10(1) 10.3390/biom10010137 PMC7022557 31947676

[60] Hanke ML, Kielian T (2011). Toll-like receptors in health and disease in the brain: mechanisms and therapeutic potential. Clin Sci (Lond).

[61] Olson JK, Miller SD (2004). Microglia initiate central nervous system innate and adaptive immune responses through multiple TLRs. J Immunol.

[62] Marinelli C (2015). Ligand engagement of Toll-like receptors regulates their expression in cortical microglia and astrocytes. J Neuroinflammation.

[63] Czapski GA, Gajkowska B, Strosznajder JB (2010). Systemic administration of lipopolysaccharide induces molecular and morphological alterations in the hippocampus. Brain Res.

[64] Creasy BM, McCoy KL (2011). Cytokines regulate cysteine cathepsins during TLR responses. Cell Immunol.

[65] Liuzzo JP, Petanceska SS, Devi LA (1999). Neurotrophic factors regulate cathepsin S in macrophages and microglia: a role in the degradation of myelin basic protein and amyloid beta peptide. Mol Med.

[66] Brown GC, Vilalta A (2015). How microglia kill neurons. Brain Res.

[67] Liu J (2008). Predominant release of lysosomal enzymes by newborn rat microglia after LPS treatment revealed by proteomic studies. J Proteome Res.

[68] Kacimi R, Giffard RG, Yenari MA (2011). Endotoxin-activated microglia injure brain derived endothelial cells via NF-kappaB, JAK-STAT and JNK stress kinase pathways. J Inflamm (Lond).

[69] Nakanishi H (2020). Cathepsin regulation on microglial function. Biochim Biophys Acta Proteins Proteom.

[70] Gonzalez-Scarano F, Baltuch G (1999). Microglia as mediators of inflammatory and degenerative diseases. Annu Rev Neurosci.

[71] Terada K (2010). Involvement of cathepsin B in the processing and secretion of interleukin-1beta in chromogranin A-stimulated microglia. Glia.

[72] Blanco P (2008). Dendritic cells and cytokines in human inflammatory and autoimmune diseases. Cytokine Growth Factor Rev.

[73] Nathan C, Xie QW (1994). Nitric oxide synthases: roles, tolls, and controls. Cell.

[74] Moss DW, Bates TE (2001). Activation of murine microglial cell lines by lipopolysaccharide and interferon-gamma causes NO-mediated decreases in mitochondrial and cellular function. Eur J Neurosci.

[75] Shen S (2005). Distinct signaling pathways for induction of type II NOS by IFNgamma and LPS in BV-2 microglial cells. Neurochem Int.

[76] Tardy C (2006). Lysosomes and lysosomal proteins in cancer cell death (new players of an old struggle). Biochim Biophys Acta.

[77] Suh HS (2009). TLR3 and TLR4 are innate antiviral immune receptors in human microglia: role of IRF3 in modulating antiviral and inflammatory response in the CNS. Virology.

[78] Cantley LC (2002). The phosphoinositide 3-kinase pathway. Science.

[79] Tas SW (2005). Signal transduction pathways and transcription factors as therapeutic targets in inflammatory disease: towards innovative antirheumatic therapy. Curr Pharm Des.

[80] Saponaro C (2012). The PI3K/Akt pathway is required for LPS activation of microglial cells. Immunopharmacol Immunotoxicol.

[81] Tsai KD (2012). Differential effects of LY294002 and wortmannin on inducible nitric oxide synthase expression in glomerular mesangial cells. Int Immunopharmacol.

[82] Kim YH (2005). LY294002 inhibits LPS-induced NO production through a inhibition of NF-kappaB activation: independent mechanism of phosphatidylinositol 3-kinase. Immunol Lett.

[83] Hazeki K (2006). Opposite effects of wortmannin and 2-(4-morpholinyl)-8-phenyl-1(4H)-benzopyran-4-one hydrochloride on toll-like receptor-mediated nitric oxide production: negative regulation of nuclear factor-{kappa}B by phosphoinositide 3-kinase. Mol Pharmacol.

[84] Avni D, Glucksam Y, Zor T (2012). The phosphatidylinositol 3-kinase (PI3K) inhibitor LY294002 modulates cytokine expression in macrophages via p50 nuclear factor kappaB inhibition, in a PI3K-independent mechanism. Biochem Pharmacol.

[85] Hu S et al (2020) PI3K mediates tumor necrosis factor induced-necroptosis through initiating RIP1-RIP3-MLKL signaling pathway activation. Cytokine 129 155046 10.1016/j.cyto.2020.155046 32114297

