# Supplementary material for: Cysteine Peptidase Cathepsin X as a Therapeutic Target for Simultaneous TLR3/4-mediated Microglia Activation
Source: Mol Neurobiol. 2022 Jan 23;59(4):2258–76. doi: 10.1007/s12035-021-02694-2 (PMC9016010; doi:10.1007/s12035-021-02694-2)
Supplement: Supplementary file 1 — Supplementary file1 (DOC 2720 KB) [file 12035_2021_2694_MOESM1_ESM.doc]

**SUPPLEMENTARY MATERIAL**

**Cysteine peptidase cathepsin X as a therapeutic target for simultaneous TLR3/4-mediated microglia activation**

Anja Pišlar ([anja.pislar@ffa.uni-lj.si](mailto:anja.pislar@ffa.uni-lj.si)),^a,^* Biljana Božić Nedeljković ([biljana@bio.bg.ac.rs](mailto:biljana@bio.bg.ac.rs)),^b^ Mina Perić ([minap@bio.bg.ac.rs](mailto:minap@bio.bg.ac.rs)),^b^ Tanja Jakoš ([tanja.jakos@ffa.uni-lj.si](mailto:tanja.jakos@ffa.uni-lj.si)),^a^ Nace Zidar ([nace.zidar@ffa.uni-lj.si](mailto:nace.zidar@ffa.uni-lj.si)),^c^ Janko Kos ([janko.kos@ffa.uni-lj.si](mailto:janko.kos@ffa.uni-lj.si))^a,d^

^a^Department of Pharmaceutical Biology, Faculty of Pharmacy, University of Ljubljana, Aškerčeva 7, SI-1000 Ljubljana, Slovenia

^b^Institute for Physiology and Biochemistry, Faculty of Biology, University of Belgrade, Studentski trg 16, 11000 Belgrade, Serbia

^c^Department of Pharmaceutical Chemistry, Faculty of Pharmacy, University of Ljubljana, Aškerčeva 7, SI-1000 Ljubljana, Slovenia

^d^Department of Biotechnology, Jožef Stefan Institute, Jamova cesta 39, SI-1000 Ljubljana, Slovenia


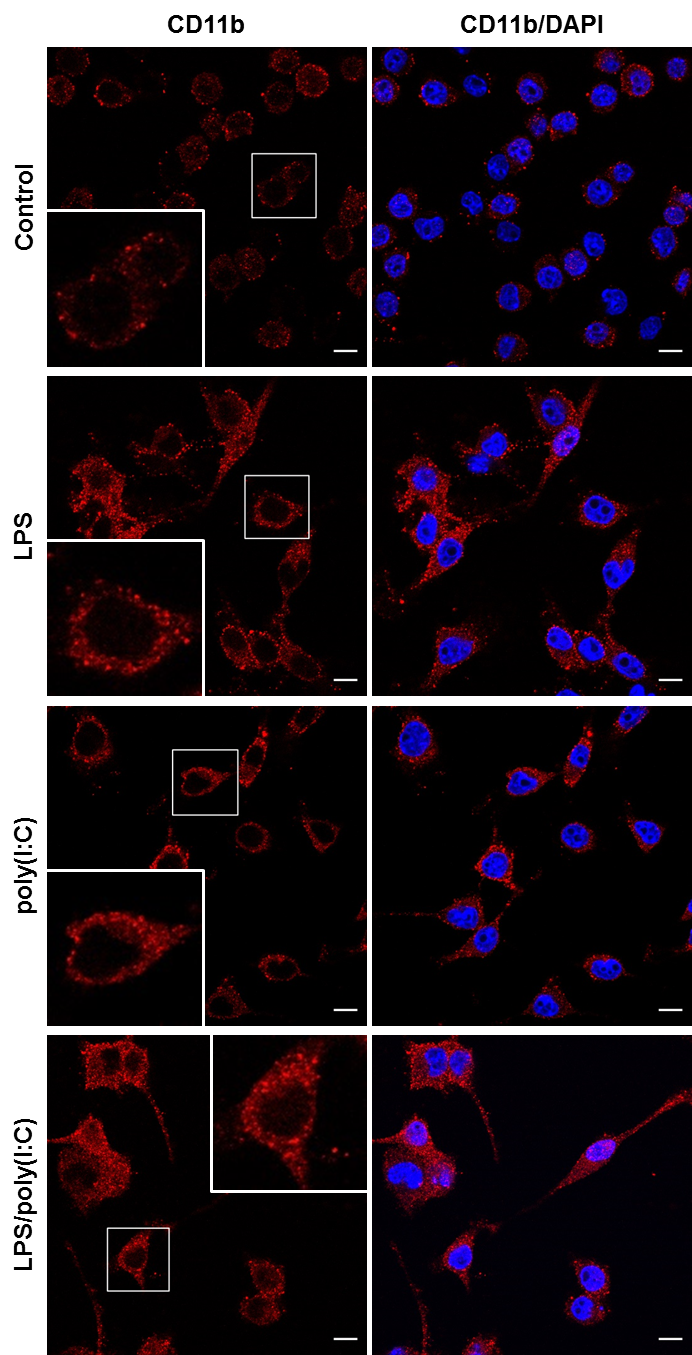


**Supplementary Figure S1. CD11b cell surface expression in activated BV2 cells.** Representative images of immunofluorescence staining of CD11b (red) in unstimulated BV2 cells (Control) and in BV2 cells stimulated with 1 µg/mL LPS and/or 10 µg/mL poly(I:C) for 24 h. Nuclei were counterstained with DAPI (blue). Scale bars: 10 µm. Insets (left): magnified images (2.5×) from the frames indicated in each panel.





**Supplementary Figure S2. Cathepsin X mRNA levels in BV2 microglia.** BV2 cells were stimulated with 1 µg/mL LPS and/or 10 µg/mL poly(I:C) in serum-free medium for the times indicated. The total RNA extracted from cells was purified and reverse transcribed. Cathepsin X mRNA levels were measured by qPCR. Data are presented as means±standard deviation of two independent experiments, each performed in duplicate. * p˂0.05.

**
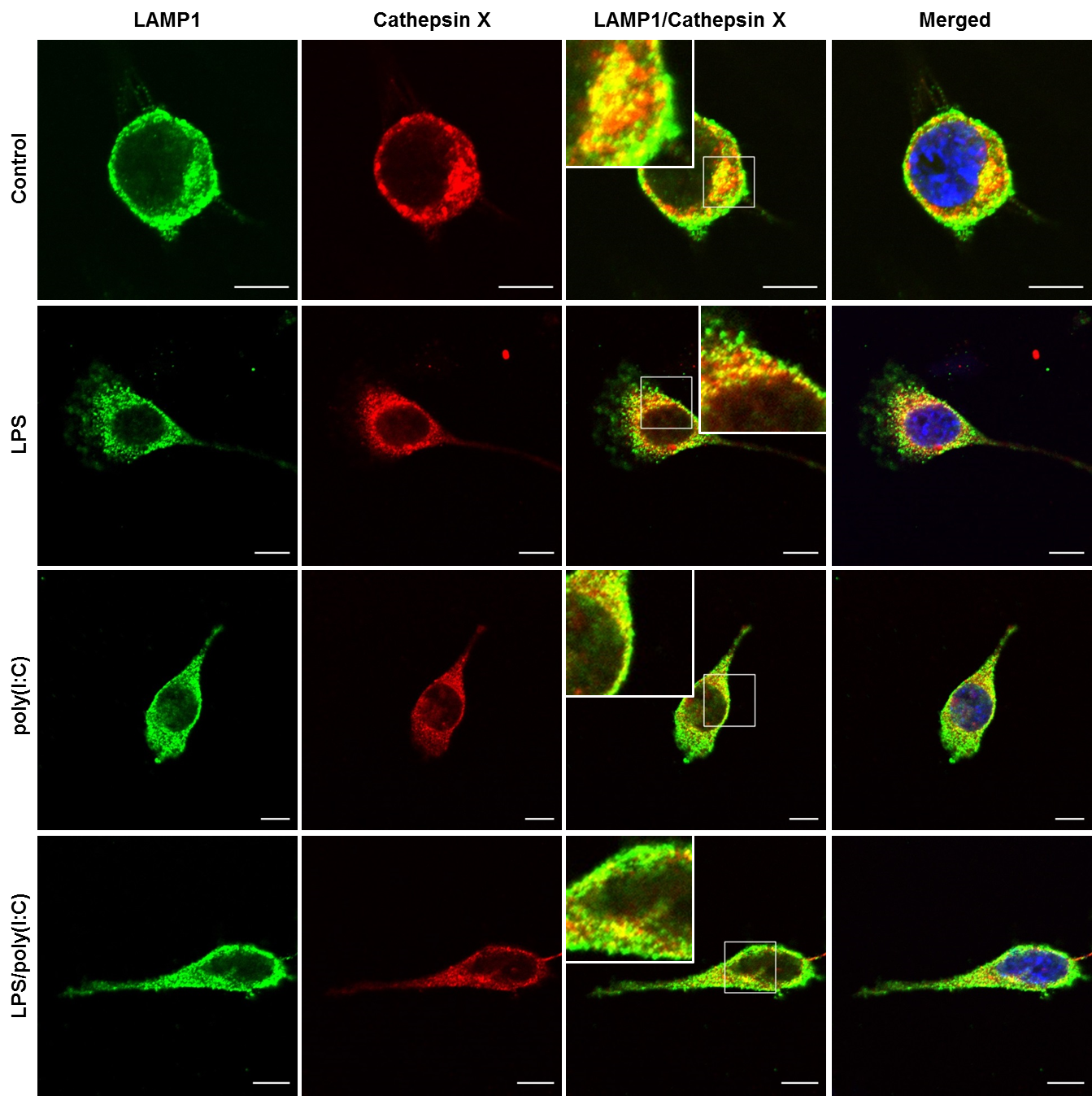
**

**Supplementary Figure S3. Lysosomal localization of cathepsin X in activated BV2 cells.** Representative images of double immunofluorescence staining of LAMP1 (green) and cathepsin X (red) in unstimulated BV2 cells (Control) and in BV2 cells stimulated with 1 µg/mL LPS and/or 10 µg/mL poly(I:C) for 24 h. Nuclei were counterstained with DAPI (blue). Scale bars: 10 µm. Insets (third column from left): magnified images (2.5×) from the frames indicated in each panel.





**Supplementary Figure S4. Cathepsin X protein levels in culture supernatants from activated primary microglia.** Primary microglia cultures were stimulated with 1 µg/mL LPS and/or 10 µg/mL poly(I:C) for 24 h. Cathepsin X protein levels in the culture supernatants of control and stimulated cells were measured with ELISA, using a goat anti-cathepsin X capture antibody (AF934) and a mouse anti-cathepsin X detection antibody (3B10). Data are presented as means±standard deviation of two independent experiments, each preformed in duplicate. ** p˂0.05.





**Supplementary Figure S5. The effect of cathepsin X down-regulation on LPS/poly(I:C)-induced NO production in microglia.** BV2 cells were transiently transfected with small-interfering RNA oligonucleotides targeting cathepsin X (siRNA-CatX) and with control siRNA (siRNA-Ctrl). After 24 h of transfection, BV2 cells were stimulated with 1 µg/mL LPS and 10 µg/mL poly(I:C) in serum-free medium for an additional 24 h. (**A**) After 48 h, cathepsin X protein levels were determined by flow cytometry. Data are expressed relative to the control and are presented as means±standard deviation of two independent experiments, each performed in duplicate. (**B**) Nitrite concentrations in the culture supernatants of the control and BV2 cells stimulated with 1 µg/mL LPS plus 10 µg/mL poly(I:C), determined using the Griess assay. Data are presented as means±standard deviation of two independent experiments, each performed in duplicate. * p˂0.05.





**Supplementary Figure S6. The effects of cysteine cathepsin inhibitors on LPS/poly(I:C)-induced microglia activation.** BV2 cells were pre-treated with the cathepsin X inhibitor AMS36 (10 µM), the cathepsin B inhibitor CA-074 (10 µM), the cathepsin S inhibitor LHVS (10 µM), and the cathepsin L inhibitor CLIK-148 (1 µM) for 1 h; this was followed by 1 µg/mL LPS plus 10 µg/mL poly(I:C) stimulation (LPS/poly(I:C)) for the indicated times. The nitrite concentrations in the collected culture supernatants of the control and stimulated BV2 cells were determined using the Griess assay. Data are presented as means±standard deviation of two independent experiments, each performed in duplicate. * p˂0.05; ** p˂0.01





**Supplementary Figure S7. The effects of the pan-caspase inhibitor zVAD-fmk on LPS/poly(I:C)-induced microglia activation.** BV2 cells were pre-treated with the pan-caspase inhibitor zVAD-fmk (10 µM), followed by 1 µg/mL LPS plus 10 µg/mL poly(I:C) stimulation for the indicated times. Cathepsin X activity in the cell lysates was determined using the cathepsin X-specific substrate Abz-Phe-Glu-Lys(Dnp)-OH. Data are presented as means±standard deviation of at least two independent experiments, each performed in duplicate.
